# Competition between electrostatic interactions and halogen bonding in the protein–ligand system: structural and thermodynamic studies of 5,6-dibromobenzotriazole-hCK2α complexes

**DOI:** 10.1038/s41598-022-23611-0

**Published:** 2022-11-08

**Authors:** Maria Winiewska-Szajewska, Honorata Czapinska, Magdalena Kaus-Drobek, Anna Fricke, Kinga Mieczkowska, Michał Dadlez, Matthias Bochtler, Jarosław Poznański

**Affiliations:** 1 Institute of Biochemistry and Biophysics PAS, Pawinskiego 5a, 02-106 Warsaw, Poland; 2 Division of Biophysics, Institute of Experimental Physics, University of Warsaw, Pasteura 5, 02-089 Warsaw, Poland; 3 International Institute of Molecular and Cell Biology, Trojdena 4, 02-109 Warsaw, Poland

**Keywords:** Biophysics, Chemical biology, Molecular biology

## Abstract

CK2 is a member of the CMGC group of eukaryotic protein kinases and a cancer drug target. It can be efficiently inhibited by halogenated benzotriazoles and benzimidazoles. Depending on the scaffold, substitution pattern, and pH, these compounds are either neutral or anionic. Their binding poses are dictated by a hydrophobic effect (desolvation) and a tug of war between a salt bridge/hydrogen bond (to K68) and halogen bonding (to E114 and V116 backbone oxygens). Here, we test the idea that binding poses might be controllable by pH for ligands with near-neutral pK_a_, using the conditionally anionic 5,6-DBBt and constitutively anionic TBBt as our models. We characterize the binding by low-volume Differential Scanning Fluorimetry (nanoDSF), Isothermal Calorimetry (ITC), Hydrogen/Deuterium eXchange (HDX), and X-ray crystallography (MX). The data indicate that the ligand pose away from the hinge dominates for the entire tested pH range (5.5–8.5). The insensitivity of the binding mode to pH is attributed to the perturbation of ligand pK_a_ upon binding that keeps it anionic in the ligand binding pocket at all tested pH values. However, a minor population of the ligand, detectable only by HDX, shifts towards the hinge in acidic conditions. Our findings demonstrate that electrostatic (ionic) interactions predominate over halogen bonding.

## Introduction

Protein kinase CK2 is a highly conserved serine/threonine kinase from the CMGC group^1^. The enzyme is ubiquitously expressed in a broad spectrum of eukaryotic cells. Unlike most other protein kinases, CK2 is constitutively active^2,3^. The holoenzyme exists in vivo as a heterotetramer consisting of two catalytic α- and/or αʹ-subunits and two regulatory β-subunits^4^. In vitro, the catalytic domain is also active as a monomeric kinase^3^. Among catalytic subunits of the holoenzyme, the α subunit has the primary role and is vital in mammalian development^5^. By contrast, the αʹ-subunit appears to be necessary only for spermatogenesis^6^. CK2 phosphorylates a diverse range of substrate proteins with serine/threonine residues in acidic regions^7^. Over 300 substrates were known in 2003^8^, and more have been discovered since. Because of the diversity of CK2 substrates, the enzyme plays a role in many medical conditions^9,10^, particularly carcinomas^11–15^. The emerging role of CK2 as an attractive cancer target has triggered the development of inhibitors. At least two of them have qualified for phase II clinical trials: CIGB-300^16–20^ and CX-4945^21–23^. Recently, inhibition of CK2 was also identified as a potential COVID-19 treatment^24,25^. CX-4945 is already in trials in this regard (https://clinicaltrials.gov/ct2/show/NCT04668209).

Many CK2 inhibitors are halogenated, including DRB^26^, brominated and iodinated benzimidazole derivatives^27–29^, and halogenated benzotriazoles^30–33^. The promising properties of halogenated benzotriazoles have prompted extensive investigations of the entire series of such inhibitors^34,35^. Halogenation alters molecular properties in several ways. It makes compounds more hydrophobic, lowers the pK_a_ of ionizable groups, and creates opportunities for halogen bonding. Halogen bonding is the favorable interaction between a halogen atom (Cl, Br, I) as the X-bond donor, and an electron-donating group as the X-bond acceptor. Halogen bonding is evidenced in structural databases by unexpectedly short interatomic contacts. Its topology is well described in protein–ligand systems: the backbone carbonyl oxygen atoms are the most abundant X-bond acceptors^36^, pi-electron systems of the peptide bond^37^ or an aromatic ring^38^ are less frequent, and sidechain oxygen atoms are identified occasionally^39^. In complexes of protein kinases with halogenated ligands, the most favored X-bond acceptors are backbone carbonyls of two residues located in the hinge region (E114 and V116 in human CK2α) and the aromatic ring of the preceding residue (F113 in hCK2α)^39,40^. The X-bond acceptor-halogen distances are shorter than the sum of their VDW radii, and the linear geometry of C-X…Acc system is strongly preferred^36^. The thermodynamic contribution of a halogen bond is still under debate^36,41–59^. However, its estimates differ substantially, varying from 0.8^60^ up to 30 kJ/mol^36^.

Many complexes of CK2 with halogenated compounds have been experimentally characterized. Such complexes are often stabilized by halogen bond(s) between the ligand and the hinge region of the protein kinase^39^. In the case of the brominated benzimidazoles or benzotriazoles, halogen bonding between ligands and the CK2 hinge comes at a price. Because of the distance between the hinge and lysine 68 (K68) in the ligand-binding pocket, halogen bonding is mutually exclusive with forming a hydrogen bond or salt bridge between the imidazole or triazole part of the ligand and the lysine amino group. The competition between alternative favorable interactions is well illustrated by a comparison of the binding modes of TBBt and its analog TBBi to maize CK2. In these complexes, TBBt chooses the salt bridge with K68^61^. By contrast, TBBi forms two halogen bonds with backbone carbonyl oxygen atoms of residues from the hinge region^62^. The competition between interactions with the hinge and K68 may depend on the protonation state of the ligand. A salt bridge to the ligand in its anionic state is expected to be more favorable than a hydrogen bond to the ligand in its neutral state. For titratable ligands with pKa values not too far from neutral, the outcome of the competition between the hinge and K68 interactions may therefore be pH-dependent.

Here, we report the thermodynamic characterization of the pH-dependent binding of titratable 5,6-DBBt (pK_a_ = 6.93^63^) to the catalytic subunit of human CK2. As a reference, we analyzed the binding of always anionic TBBt (pK_a_ ~ 5^64^) and four non-ionizable neutral analogs: 1-CH_3_- and 2-CH_3_-derivatives of 5,6-DBBt and TBBt. We present structures of hCK2α in complex with either 5,6-DBBt or TBBt, determined for crystals grown at pH ranging from 5.5 up to 8.5. The structural data are complemented by hydrogen–deuterium exchange experiments performed for hCK2α in a complex with 5,6-DBBt at two extreme pH values (6.7 and 8.7). Presented results explicitly confirm that the direct electrostatic interactions predominate over a possible contribution of halogen bonding.

## Results

### pH-dependent binding of “conditionally anionic” 5,6-DBBt and “constitutively anionic” TBBt and their non-dissociable 1- and 2-methyl analogs monitored by low-volume Differential Scanning Fluorimetry (nanoDSF)

We have used low-volume fluorescence-monitored thermal denaturation (nanoDSF) to assess the contribution of the electrostatic interactions and the ionization state of the free ligand to the binding affinity of 5,6-DBBt to hCK2α. We have analyzed the thermal stability of hCK2α alone and in the presence of tenfold excess of ligands: 5,6-DBBt, its non-dissociable derivatives (1-CH_3_-5,6-DBBt, 2-CH_3_-5,6-DBBt), anionic TBBt, and its neutral non-dissociable derivatives (1-CH_3_-TBBt and 2-CH_3_-TBBt). We have previously published the semi-quantitative analysis of hCK2α binding affinities to variously brominated benzotriazoles^34,35^, including 5,6-DBBt and TBBt. In this work, we used TBBt (pK_a_ = 4.8 ± 0.4; 4.78^63^, 5^64^) as a consistently anionic ligand in the tested pH range and the compounds with a methyl group attached to the triazole ring as ligands that remain neutral under the experimental conditions. This approach allows formal separation of the purely electrostatic contribution resulting from the dissociation of the triazole proton from other effects.

**Figure 1:**
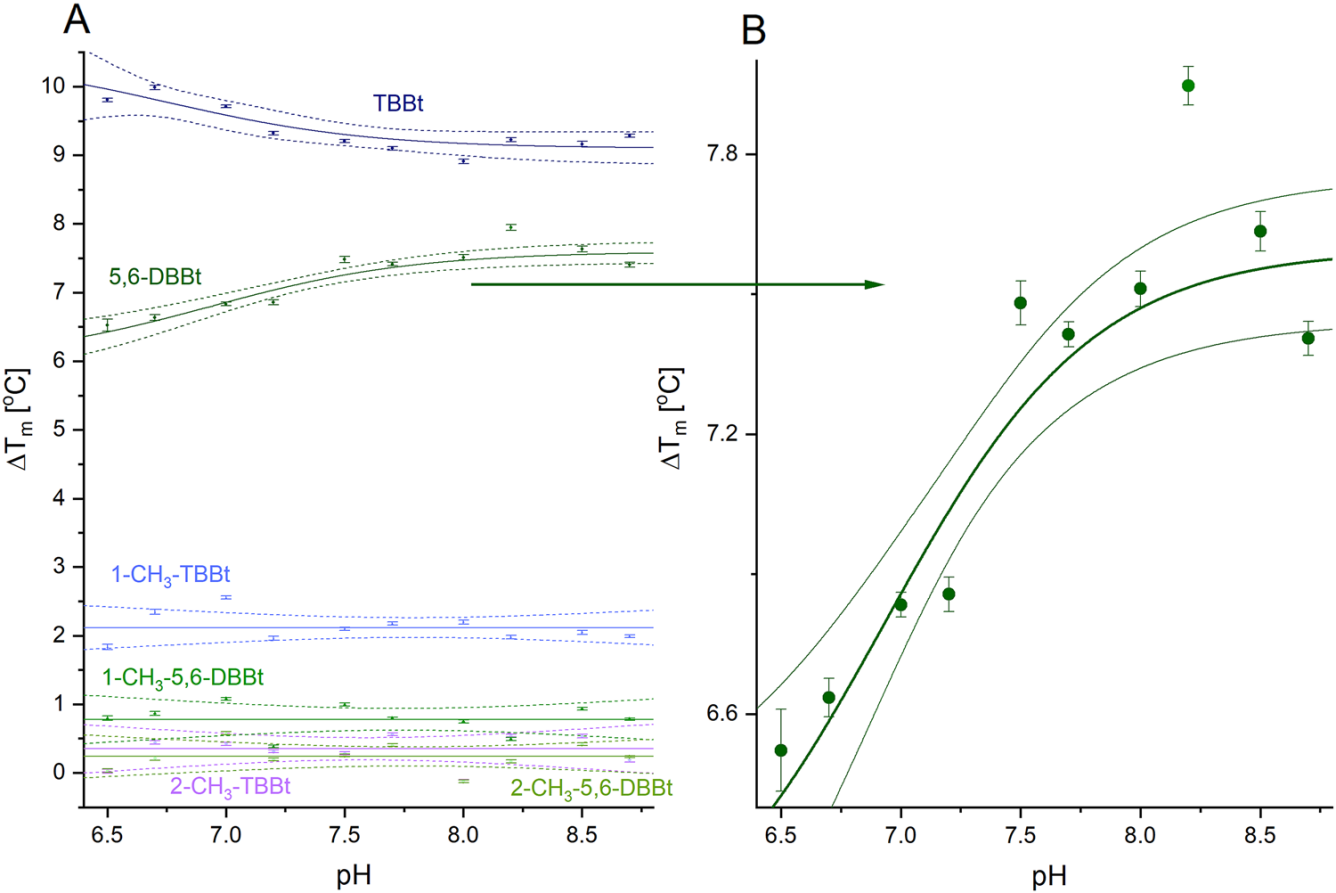
pH-dependence of ΔT_m_ for the complexes of hCK2α with 5,6-DBBt, TBBt, and their non-dissociable analogs determined using nanoDSF. Comparison of all tested compounds (**A**), results for 5,6-DBBt at a different scale (**B**).

The mid-point temperature of the thermal denaturation of hCK2α complexes, T_m_, is substantially increased relatively to the free enzyme: at pH 8.7 by 7.2 and 9.3 °C for 5,6-DBBt and TBBt, respectively (Table 1, Fig. 1A). According to the Fisher test, at the significance level of 0.05, the changes in ΔT_m_ in the presence of 5,6-DBBt must be regarded as pH-dependent (Fig. 1B), roughly reflecting the expected change in the protonation equilibrium of the ligand (pKa = 6.93^63^). An inverse trend of pH-dependent changes observed for TBBt must indicate an alternative source of variation in the apparent binding affinity, presumably associated with the ionization of a neighboring histidine (H160, theoretical pK_a_ ~ 7). Its cationic form possibly enhances the binding of the anionic ligand under moderately acidic conditions. This mechanism is supported by the structure of hCK2α with TBBt (PDB 6TLL^65^), in which the imidazole ring of H160 is close to the triazole ring of TBBt, with the shortest distance between multiple locations of these rings of 3.7 Å (Suppl. Fig. S1).

**Table 1:** Thermal stability of hCK2α in the free form and in the presence of tenfold excess of bromobenzotriazole ligands as assessed by nanoDSF.

pH	Free hCK2α	TBBt	1-CH_3_-TBBt	2-CH_3_-TBBt	5,6-DBBt	1-CH_3_-5,6-DBBt	2-CH_3_-5,6-DBBt
	T_m_ [°C]	ΔT_m_ [°C]					
6.5	41.70 ± 0.01	9.80 ± 0.04	1.83 ± 0.04	0.02 ± 0.03	6.52 ± 0.09	0.79 ± 0.03	0.03 ± 0.03
6.7	42.38 ± 0.01	9.99 ± 0.03	2.35 ± 0.03	0.44 ± 0.03	6.64 ± 0.04	0.87 ± 0.03	0.21 ± 0.03
7.0	43.65 ± 0.01	9.71 ± 0.02	2.55 ± 0.02	0.42 ± 0.02	6.83 ± 0.03	1.08 ± 0.02	0.58 ± 0.03
7.2	44.94 ± 0.01	9.32 ± 0.03	1.96 ± 0.03	0.32 ± 0.02	6.86 ± 0.04	0.38 ± 0.02	0.20 ± 0.02
7.5	45.44 ± 0.01	9.20 ± 0.02	2.10 ± 0.02	0.29 ± 0.02	7.48 ± 0.05	1.00 ± 0.02	0.26 ± 0.02
7.7	46.11 ± 0.01	9.10 ± 0.03	2.18 ± 0.02	0.56 ± 0.02	7.41 ± 0.03	0.79 ± 0.02	0.41 ± 0.02
8.0	46.33 ± 0.01	8.91 ± 0.03	2.20 ± 0.03	− 0.12 ± 0.02	7.51 ± 0.04	0.75 ± 0.02	− 0.13 ± 0.02
8.2	46.38 ± 0.01	9.23 ± 0.03	1.98 ± 0.03	0.54 ± 0.02	7.95 ± 0.04	0.49 ± 0.02	0.17 ± 0.02
8.5	46.24 ± 0.02	9.16 ± 0.04	2.05 ± 0.03	0.54 ± 0.03	7.63 ± 0.04	0.93 ± 0.02	0.42 ± 0.02
8.7	46.23 ± 0.01	9.28 ± 0.02	1.99 ± 0.02	0.19 ± 0.03	7.41 ± 0.04	0.78 ± 0.02	0.24 ± 0.02

1-CH_3_-5,6-DBBt and 1-CH_3_-TBBt, the asymmetric non-dissociable derivatives of 5,6-DBBt and TBBt, affect the protein stability much less: their binding increases the melting temperature by 1.8–2.6 °C and 0.4–1.1 °C, respectively (Table 1, Fig. 1A), indicating that the anionic form is highly preferred for both 5,6-DBBt and TBBt. In the published PDB structures (3OFM, 3RPS, 6HMQ, 7AT9) with bigger alkyl substitutions at N1 of TBBt, the steric bulk in this position does not significantly perturb the interaction with the ATP-binding site of human protein kinase CK2α/CK2αʹ^66–68^. This, together with modeling (Suppl. Fig. S2A,B), confirms that possible steric effects are negligible. However, methylation at N1 makes the benzotriazole derivatives neutral, which interferes with ligand interaction with K68 and possibly affects the water network at the binding site. Thus, it must be concluded that, at least in the studied system, direct electrostatic interactions predominate over other types of interactions, including putative halogen bonding.

At the significance level of 0.05, the ligand-induced changes in ΔT_m_ remain pH-independent for the two neutral ligands, but small perturbations at lower pH observed for 1-CH_3_-TBBT reproduce the trend identified for TBBt. The hydrophobic effect and halogen bonding can explain the slight difference in binding affinity between 1-CH_3_-TBBt and 1-CH_3_-5,6-DBBt because the ligand carrying more bromine atoms is much more hydrophobic and possibly forms more halogen bonds. The binding affinity of 1-CH_3_-5,6-DBBt was independently estimated by ITC, indicating the K_diss_ of 1.56 ± 0.52 μM (weighted average from Suppl. Table S1).

Both 2-CH_3_-derivatives do not stabilize the protein at all tested pH values (ΔT_m_ does not exceed 0.5 °C). Lack of stabilization indicates that these ligands do not interact with hCK2α. In silico modelling of hCK2α in complex with 2-CH_3_-5,6-DBBt (Suppl. Fig. S2C) confirms that the methylation of benzotriazole at N2 introduces steric hindrances and the binding poses of N2-methylated derivatives must differ from those observed for unsubstituted ones.

### The pH-dependent binding affinity of 5,6-DBBt monitored with ITC

**Figure 2:**
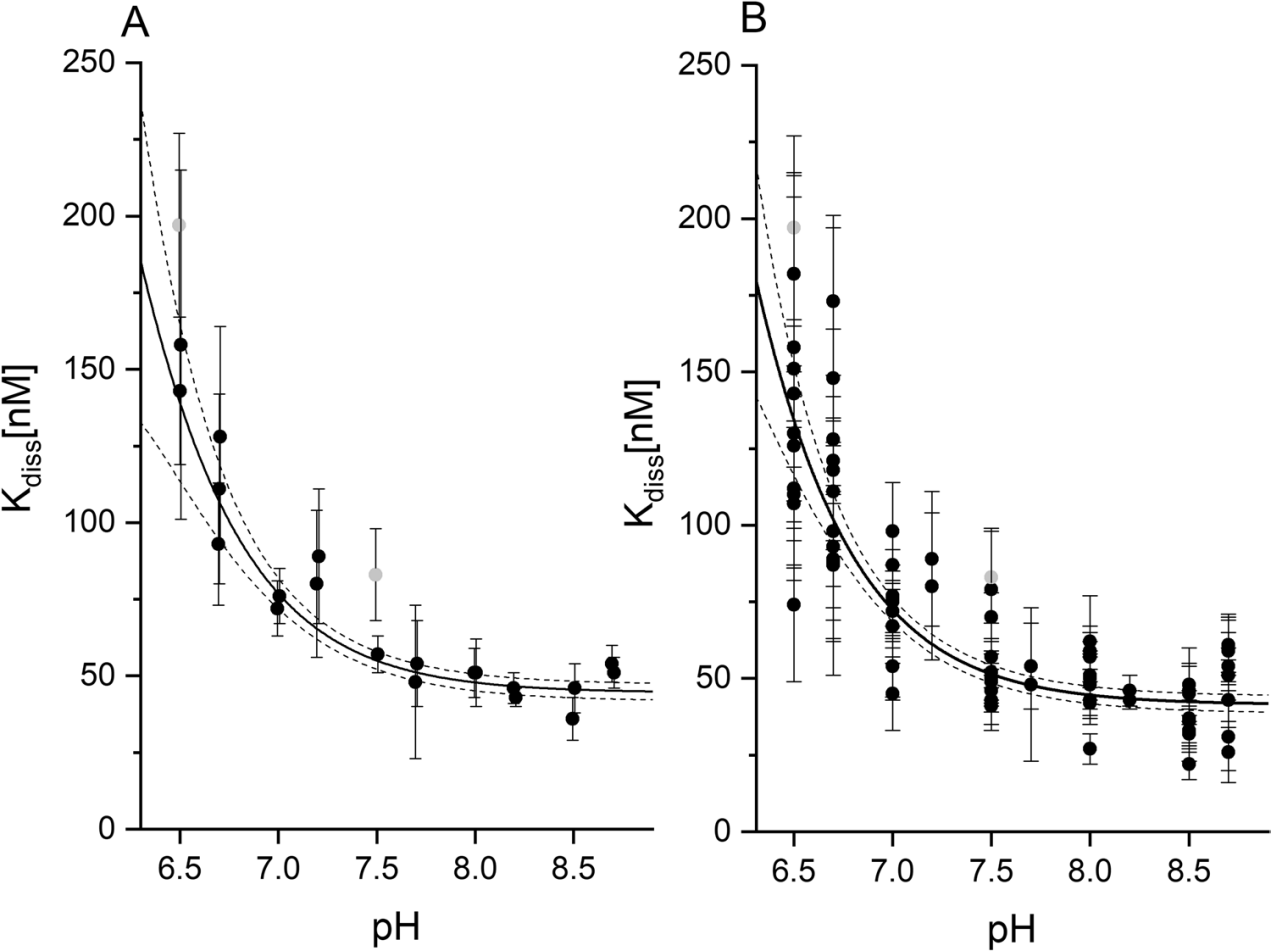
pH-dependence of binding of 5,6-DBBt to the hCK2α. ITC-derived K_diss_ values were estimated solely in Bis–Tris Propane buffer (**A**) or using a set of eleven buffers at each pH chosen according to the pH-dependent buffering properties (**B**).

The binding of 5,6-DBBt to hCK2α was initially studied in the pH range of 6.5 to 8.7 in Bis–Tris Propane buffer using isothermal titration calorimetry (ITC). The resulting thermodynamic parameters are summarized in Table 2 and presented in detail in Suppl. Table S1. The apparent binding affinity to hCK2α varies non-linearly (Fig. 2A), with the putative inflection point roughly reflecting the pK_a_ for dissociation of the 5,6-DBBt triazole proton (6.93^63^).

**Table 2:** Thermodynamic parameters for hCK2α-5,6-DBBt interaction determined by ITC.

pH	ΔG [kJ·mol^−1^]*	Kd [nM]*	n_H+_
6.5	− 38.7 ± 0.3	163 ± 18	− 0.68 ± 0.18
6.7	− 39.8 ± 0.4	104 ± 15	− 0.47 ± 0.16
7.0	− 40.7 ± 0.2	74 ± 7	− 0.39 ± 0.04
7.2	− 40.4 ± 0.5	85 ± 16	–
7.5	− 41.1 ± 0.2	61 ± 6	− 0.20 ± 0.03
7.7	− 41.5 ± 0.6	53 ± 12	–
8.0	− 41.6 ± 0.3	51 ± 7	− 0.02 ± 0.03
8.2	− 42.0 ± 0.2	44 ± 3	–
8.5	− 42.1 ± 0.3	40 ± 5	− 0.04 ± 0.09
8.7	− 41.6 ± 0.2	52 ± 4	0.01 ± 0.08

*Weighted averages calculated from data presented in Suppl. Table. S1.

The neutral form of 5,6-DBBt predominating at lower pH binds to hCK2α visibly weaker than the anionic one at pH 8.7, while the hydrophobic solvation should favor the binding of the neutral ligand. This tendency could be explained by different binding modes of the neutral and anionic forms of the ligand. However, it is most likely associated with binding-induced ligand deprotonation. The thermodynamic contribution of such a process can be described by pH-dependent change of the ligand-binding affinity according to Eq. (1)^69^:

1 $${K}_{diss}={K}_{int}\cdot\frac{1+{10}^{{(pK}_{a}^{f}-pH)}}{1+{10}^{{(pK}_{a}^{c}-pH)}}$$

where K_int_ is the intrinsic dissociation constant for the anionic form of the ligand, pK_a_^f^, and pK_a_^c^ are the pK_a_ values for deprotonation of this ligand in solution and in complex with hCK2α, respectively. The model was fitted to the experimental K_diss_ values determined at varying pH. pK_a_^f^ was constrained to the experimental value of 6.93^63^, while the values of pK_a_^c^ and K_int_ were fitted using the experimental data (5.6 ± 0.4 and 44.3 ± 1.3 nM, respectively). The change in the protonation state of the ligand contributes significantly to its binding to hCK2α. For the neutral form of 5,6-DBBt (i.e., when ${10}^{({pK}_{a}^{f}-pH)} \gg 1)$ K_diss_ can be, according to Eq. (1), roughly estimated as ${K}_{int}\cdot{10}^{{(pK}_{a}^{f}-{pK}_{a}^{c})}$ ≈ 1 μM, which is close to the experimental value determined for 1-CH_3_-5,6-DBBt (1.56 ± 0.52 μM).

The thermodynamic parameters for 5,6-DBBt-hCK2α binding were determined at seven pH values in the range of 6.5–8.7 in eleven buffered solutions (Suppl. Material), each characterized by the different enthalpy of ionization^70^. We have confirmed the absence of buffer-specific effects since the K_diss_ vs. pH relationship for multiple buffering solutions agrees with that determined solely in the Bis–Tris Propane (Fig. 2B).

### Protonation balance upon ligand binding assessed from buffer-dependent ITC-derived heat of binding

In general, any binding associated protonation event affects the apparent enthalpy of ligand binding, ∆*H*_*bind*_, by the individual contribution of ionization enthalpy of the particular buffer, $\Delta {H}_{i}^{b}$, according to the formula:

2 $$\Delta {H}_{bind}=\Delta {H}_{0}+\Delta {H}_{i}^{b}\cdot{\mathrm{n}}_{{H}^{+}}$$

where n_*H*+_ describes the number of protons transferred from the buffer upon forming the protein–ligand complex^69^.

**Figure 3:**
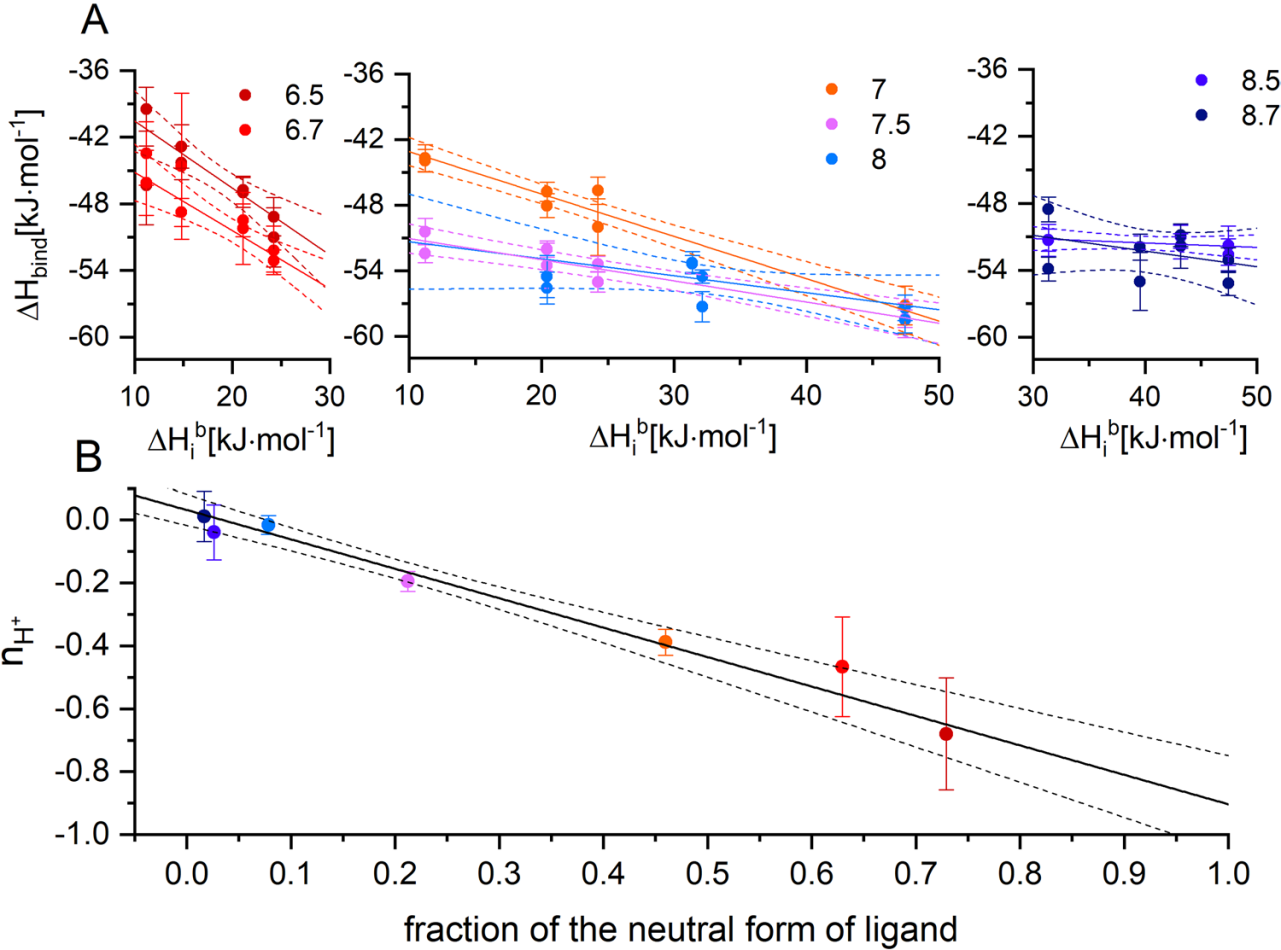
pH dependence of the heat of 5,6-DBBt-hCK2α binding determined in various buffered solutions characterized by different heats of buffer ionization (**A**). The negative slope at moderately acidic conditions indicates proton release upon complex formation. Correlation between the estimated number of transferred protons—n_H+_ and the pH-dependent dissociation equilibrium of the free 5,6-DBBt (**B**).

We have applied Eq. (2) to the ITC data collected at various pH values (6.5, 6.7, 7.0, 7.5, 8.0, 8.5, and 8.7) in eleven different buffers (Fig. 3A). At basic conditions (pH ≥ 8), ∆*H*_*bind*_ does not depend on $\Delta {H}_{i}^{b}$, clearly proving that the monoanionic form of the ligand, which dominates in solution, also predominates when 5,6-DBBt binds to the protein. However, the buffer contribution at lower pH becomes significant with negative n_*H*+_ values (Table 2). According to the F-test (F(3,37) = 27.17, p < 10^–11^), the model with pH-dependent n_*H*+_ must be regarded better than that with a universal pH-independent n_*H*+_ value. The variation in the apparent number of transferred protons, n_*H*+_, correlates with the pH-dependent dissociation equilibrium of the free 5,6-DBBt (Fig. 3B), unequivocally proving that the observed change of binding affinity with pH must be attributed to the binding-induced deprotonation of the ligand. The data indicate that a proton is released to the buffered solution upon ligand binding at moderately acidic conditions. Therefore, the ligand in the anionic form is bound, even at lower pH.

It is worth emphasizing that the term ∆*H*_0_ in Eq. (2) contains not only the intrinsic binding enthalpy, $\Delta {H}_{intr}$, but also additional contributions associated with protonation events^69^:

3 $$\Delta {H}_{0}=\Delta {H}_{intr}+{\overline{H} }^{f}\cdot\Delta {H}_{p}^{f}+{\overline{H} }^{c}\cdot\Delta {H}_{p}^{c}$$

where ${\overline{H} }^{f},\Delta {H}_{p}^{f}$, and ${\overline{H} }^{c},\Delta {H}_{p}^{c}$ are the fraction of protons and the enthalpy of ligand protonation in solution and in the complex, respectively. For a single protonation event, the above equation could be rephrased as^69^:

4 $${\Delta H}_{\mathrm{bind}} ={H}_{intr}+({\Delta H}_{i}-{\Delta H}_{diss})\cdot\ \Delta \mathrm{n }+{\Delta \Delta H}_{diss}\cdot\frac{{10}^{{(pK}_{a}^{c}-pH)}}{{1+10}^{{(pK}_{a}^{c}-pH)}}$$

where $\Delta \mathrm{n}=\frac{1}{1+{10}^{({pK}_{a}^{f}-pH)}}-\frac{1}{1+{10}^{({pK}_{a}^{c}-pH)}}$ is the net proton transfer upon a binding event, and ${pK}_{a}^{c}$ and ${pK}_{a}^{f}$ are the ${pK}_{a}$ values for proton dissociation for the ligand in complex and in solution, respectively. ∆*H*_*i*_ and ∆*H*_*diss*_ are the heats of (de)protonation for the buffer and free ligand, and ∆∆*H*_*diss*_ is the change of the heat of (de)protonation for the ligand bound to the protein.

**Figure 4:**
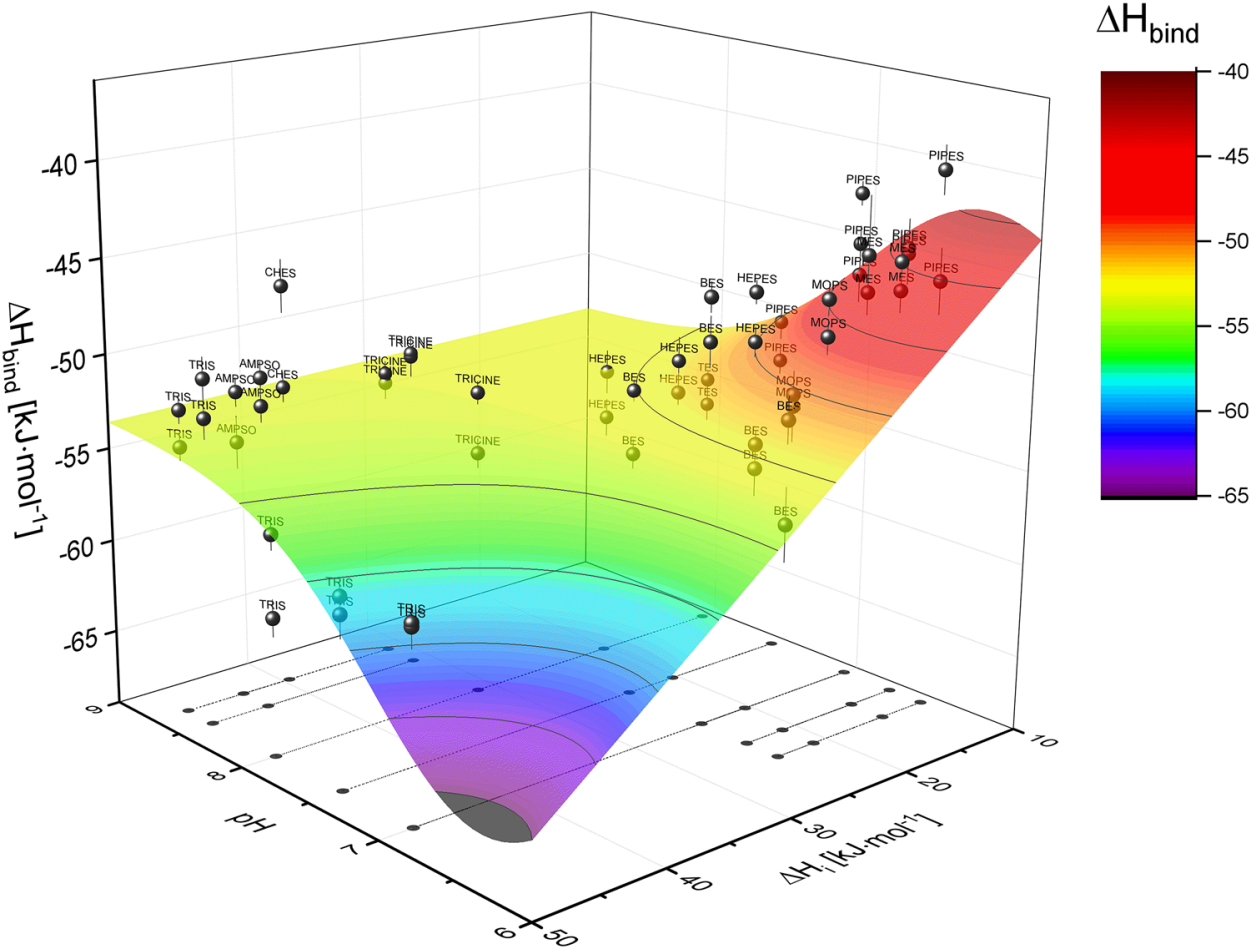
Heat of 5,6-DBBt binding to hCK2α determined in various conditions. The surface, representing the model of the binding-induced shift of ligand ionization [Eq. (4)], is colored according to the heat of binding. Each data point, representing an individual titration experiment, is accompanied by a standard error (vertical line) and denoted by the name of the buffer used in the experiment.

Global fitting of the experimentally determined ∆*H*_*bind*_ as a function of pH and ∆*H*_*i*_ [Eq. (4)] leads to estimates of all parameters connected with the deprotonation event. A fitted three-dimensional surface relating the experimental ∆*H*_*bind*_ to pH and ∆*H*_*i*_ is shown in Fig. 4. The pK_a_ values estimated from the analysis equal 6.99 ± 0.16 for the free ligand and 6.1 ± 0.3 for the ligand in the complex. Both are close to the values estimated from the *K*_*diss*_ data. The intrinsic enthalpy, ∆*H*_*intr*_, is – 53.7 ± 0.5 kJ·mol^−1^, while the enthalpy of protonation in the free state, ∆*H*_*diss*_, is − 29 ± 5 kJ·mol^−1^, and upon binding changes to − 21 ± 14 kJ·mol^−1^ (∆∆*H*_*diss*_ ~ 8 kJ·mol^−1^).

### Structure determination

The structures of 5,6-DBBt and TBBt in complex with hCK2α obtained at pH 7.5 were published within a set of eight bromobenzotriazole-hCK2α complexes^65^. In order to provide the structural interpretation of the thermodynamic data, we have now grown crystals of human CK2α in complex with either 5,6-DBBt or TBBt in the pH range of 5.5–9.5. 5,6-DBBt-hCK2α complex crystallized up to pH 8.5, whereas TBBt crystals were obtained only at pH 7.5^65^ and 8.5 (Table 3). The electron densities indicate that the ligands are located in the ATP binding pocket of the kinase. 5,6-DBBt adopted a single pose highly similar for all pH values. For TBBt, multiple poses were observed at both pH values, but the weaker ones were less prominent at pH 8.5, possibly due to the lower resolution of the crystals. In some cases, we collected data for several crystals to confirm that the ligand binding poses and the results were internally consistent (Suppl. Table S2 and Suppl. Fig. S3).

**Table 3:** Data collection and refinement statistics.

	hCK2-5,6-DBBt	hCK2-5,6-DBBt	hCK2-5,6-DBBt	hCK2-4,5,6,7-TBBt
pH	**5.5**	**6.5**	**8.5**	**8.5**
Inhibitor	co-crystallized	co-crystallized	soaked	soaked
Mg^2+^	4 mM	4 mM	4 mM	4 mM
**Data collection**				
Space group	*P*4_2_2_1_2	*P*4_2_2_1_2	*P*4_2_2_1_2	*P*4_2_2_1_2
Unit cell dimensions				
a, b (Å)	127.5	128.5	129.5	127.8
c (Å)	61.0	61.2	60.9	61.2
Wavelength (Å)	0.9117	0.9117	0.9117	0.9117
Beamline	DESY P11	DESY P11	DESY P11	DESY P11
Resolution (Å)	**2.55**	**2.58**	**2.30**	**2.27**
Lowest shell	(45.1–7.55)	(45.4–7.64)	(45.8–6.84)	(45.2–6.74)
Highest shell	(2.70–2.55)	(2.73–2.58)	(2.44–2.30)	(2.41–2.27)
R_meas_ (%)*	**30.4** (9.4, 135.9)	**31.4** (6.2, 210.5)	**11.2** (3.9, 87.3)	**11.5** (3.6, 174.9)
CC_1/2_*	**99.5** (99.9, 80.3)	**99.6** (99.9, 68.6)	**99.9** (100, 93.5)	**100** (100, 84.5)
I/σI*	**10.7** (31.7, 2.03)	**12.6** (43.4, 1.99)	**27.7** (80.2, 4.24)***	**26.1** (88.4, 2.53)***
Completeness (%)*	**99.9** (99.3, 99.8)	**99.8** (99.3, 99.8)	**99.2** (99.5, 99.4)	**99.8** (99.5, 99.5)
Multiplicity*	**26.3** (23.0, 26.9)	**26.0** (25.3, 26.4)	**25.4** (22.3, 26.1)	**26.3** (21.9, 26.5)
Number of reflections	16,932	16,669	23,379	24,013
**Refinement**				
R_work_	17.05	17.99	17.91	17.12
R_free_	23.15	23.44	22.06	20.54
No. atoms**	3448	3263	3386	3519
Protein	3087	2886	2848	3111
Ligand	11	22	22	26
Other	350	269	385	382
Bond lenghts rmsd (Å)	0.007	0.007	0.007	0.007
Bond angles rmsd (°)	1.18	1.17	1.19	1.22
Ramachandran				
Allowed (%)	100	100	100	100
Favored (%)	95.8	96.4	97.3	96.7
Molprobity clashscore	1	1.0	1.5	0.5
PDB accession code	7QGC	7QGB	7QGD	7QGE

Significant values are in bold.

The statistics for crystal replicates are presented in Suppl. Table S2.

*Lowest and highest shell in brackets. **Alternative conformations counted separately. ***Data cut due to ice rings.

The hydrophobic faces of 5,6-DBBt stack on one side against V53 and V66 of the N-terminal lobe and on the other side against M163 and I174 from the C-terminal lobe. The phenyl ring of F113, tilted relative to the benzotriazole ring, may limit the penetration depth of the ligand into the pocket. In the plane of the benzotriazole ring, the ligand position is predominantly determined by the salt bridge between the N2 of 5,6-DBBT and the ε-amino group of K68. The bromine atoms of 5,6-DBBt are pointed roughly towards the mainchain oxygen atoms of E114 and N117 and the sidechain carbonyl oxygen atom of N118. However, in all cases, bromine-oxygen distances are too large for halogen bonding (Suppl. Table S3). The predominance of salt bridge formation over halogen bonding at all pH values is also supported by the composite omit maps and anomalous diffraction data, making it possible to accurately locate the bromo substituents (Suppl. Figs. S3 and S4).

**Figure 5:**
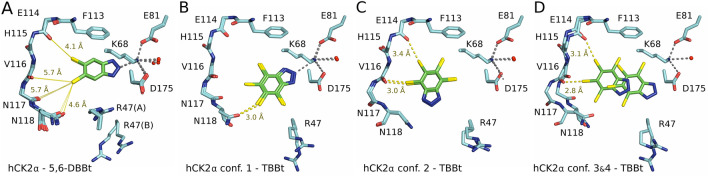
Crystal structures of brominated benzotriazoles in complex with hCK2α. The structures determined for crystals grown at various pH values have been overlaid. The single binding pose of 5,6-DBBt is consistent across the 5.5–8.5 pH range (**A**). Two dominant orientations of TBBt are observed at both pH values (7.5 and 8.5) (**B**, **C**), weaker binding poses of TBBt are only observed at pH 7.5^65^ (**D**). Protein carbon atoms are in light blue, and benzotriazole carbon atoms are in green. Bromo substituents and their distances to carbonyl oxygens are in yellow.

The crystallographic data indicate significant differences between the binding modes of 5,6-DBBt and TBBt. 5,6-DBBt is bound in a single, pH-independent pose, compatible with salt bridge formation of the (anionic) ligand, but not halogen bonding. By contrast, TBBt is bound in multiple poses, some of which feature halogen bonds. The weaker poses of TBBt are not observed at pH 8.5, but it might be due to the weaker diffraction of the crystals. Due to steric constraints, the binding mode of 5,6-DBBt is not anticipated by any of the binding modes of TBBt. Compared to TBBt, 5,6-DBBt is bound more “deeply” in the pocket. TBBt could not bind in this pose because bromo substituents in the 4- and 7-positions would clash with F113 and I95. Moreover, the dominant conformation of R47 in the complex with 5,6-DBBt would also preclude the experimentally observed binding mode of TBBt. In the complex with TBBt, R47 gives way, so the bulkier TBBt fits into the pocket (Fig. 5, Suppl. Figs. S3 and S5).

### Ligand-induced changes in hydrogen/deuterium exchange monitored by mass spectrometry (HDX-MS)

HDX-MS experiments could indirectly reveal the ligand pose in the complex because the internal dynamics of the hinge region is expected to be affected by the ligand location. Hence, we compared the position of 5,6-DBBt in complex with hCK2α at pH 6.7 and 8.7. At both conditions, the deuterium exchange for the free form of hCK2α and its complex with 5,6-DBBt was monitored after 10 s exposure to D_2_O.

**Figure 6:**
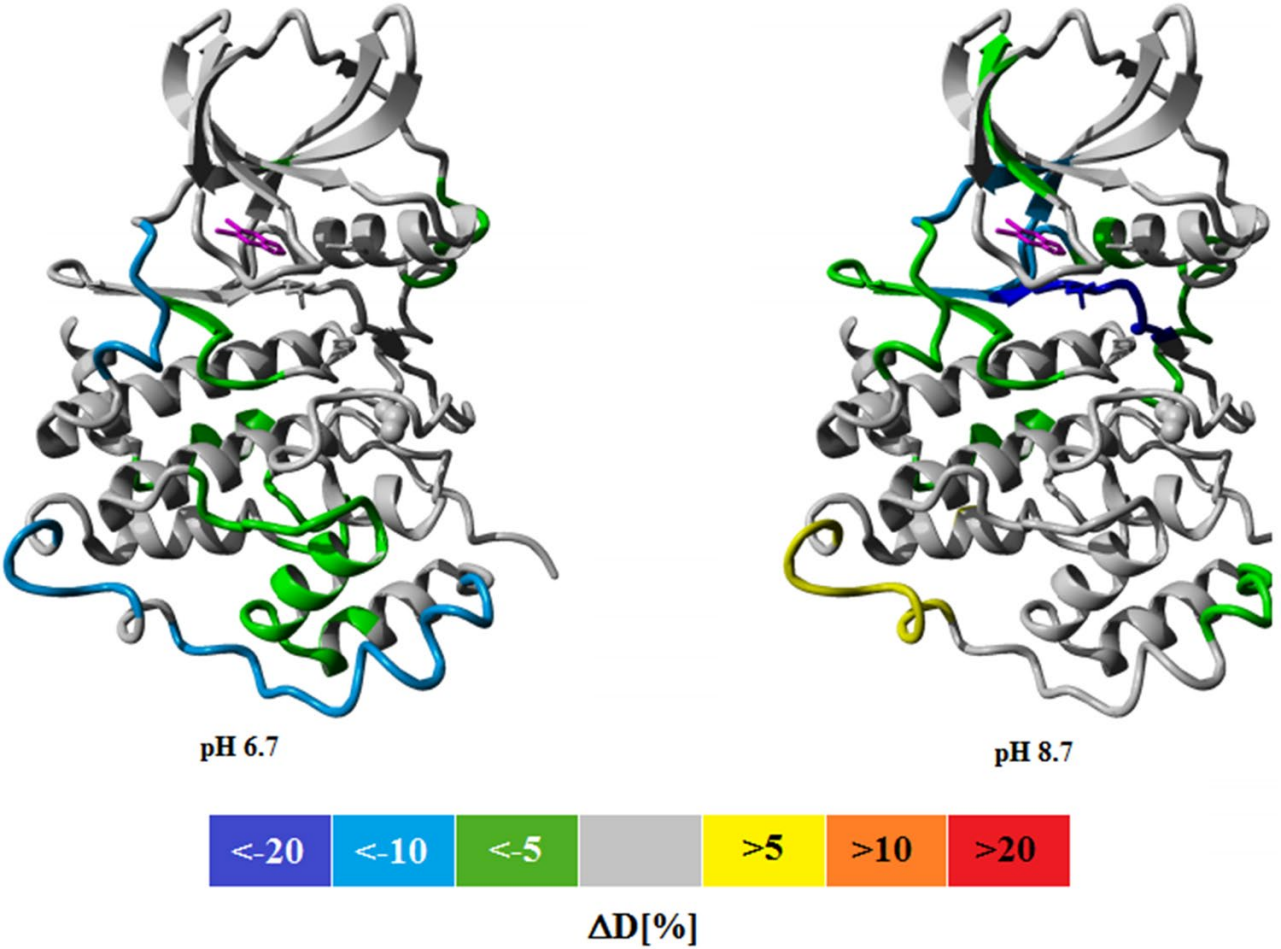
Color-coded representation of the differences in the deuteration of hCK2α due to 5,6-DBBt binding at pH 6.7 and 8.7 (10 s hydrogen deuterium exchange applied).

Overall, 5,6-DBBt protected the ATP binding site better at pH 8.7 than at pH 6.7 due to the higher affinity of the ligand at a basic pH (Fig. 6). The highest ligand-induced protection, exceeding 20%, was observed at pH 8.7 for residues R172-E180, covering the DWG motif of the activation loop. No statistically significant protection was seen for this region at lower pH. At pH 8.7, the protection was also seen for regions covering residues V112-V116 and G52-N58 (Fig. 6). The effect can be attributed to a second binding site, which is occupied in some of the structures of the complex with 5,6-DBBt and in complex with TBBt (Suppl. Table S2, Suppl. Fig. S6 and ^65^) and confirmed for 5,6-DBBt by MST data^71^.

Against the overall better protection of the ATP binding pocket at higher pH due to tighter ligand binding, one fragment (residues N117-L124) corresponding to the hinge region of the kinase was better protected by 5,6-DBBt at lower pH (H/D exchange reduced by 10% at pH 6.7, but only by 5% at pH 8.7). This effect is consistent with an expected pH-driven shift in the ligand pose. At higher pH, 5,6-DBBt binds closer to K68 (anchored by salt bridges to E81 and D175, shown in stick representation in Fig. 6). As a consequence, it is away from the hinge and provides minimal protection to this region. The data suggest that at lower pH the ligand may move towards the hinge. It could be due to a slight pose change of the majority of ligand molecules in the population. Alternatively, a minor fraction of the ligands may shift more substantially and favor halogen bonding to the hinge over the interaction with K68. The protonation equilibrium is faster than the ligand shift, so at a lower pH, the ligand is statistically closer to the hinge. However, only the dominant pose is visible in the crystal structures.

Ligand binding also induced global changes in internal dynamics/flexibility of hCK2α. The binding of 5,6-DBBt at pH 6.7 slowed down hydrogen–deuterium exchange for numerous residues located in the C-terminal lobe of the GHI subdomain, especially for the CMGC kinase-specific α-GH insert (L249-Q290, Fig. 6). This region seems irrelevant to the structural stability of the C-terminal domain, but it is highly conserved throughout the whole CMGC group and putatively involved in the substrate recognition^72,73^.

## Discussion

Recent studies have suggested that the ligand-binding pose of halogenated benzimidazoles and benzotriazoles in EPKs is determined by a competition between hydrogen bonding/salt bridge formation and halogen bonding^61,62^. In this tug of war, halogen bonding pulls the ligands towards the hinge region. Conversely, hydrogen bonding/salt bridge formation drags them towards K68 (the lysine from the N-lobe involved in binding of the α- and β-phosphate in the productive complex with the co-substrate ATP), favoring the ligand binding pose further away from the hinge. The outcome of this competition is difficult to predict, in part because the thermodynamic contribution of halogen bonding to intermolecular interaction remains controversial, with free energy estimates for aqueous media ranging from 0.2^60^ up to 7^36^ kcal/mol. Moreover, the shape and hydrophobicity modulate ligand poses in the kinase pocket.

5,6-DBBt with pK_a_ = 6.93 is neutral at low and anionic at high pH. In maize CK2, we observed two 5,6-DBBt poses, one with halogen bonding to the hinge and another with salt bridge formation to K68 (5TS8^74^). Assuming that halogen bonds may outcompete a hydrogen bond but not a salt bridge, we reasoned that the binding pose of 5,6-DBBt may be pH-controllable. H/D exchange data support the idea. Mainchain amide protons in the hinge region are better protected at low than high pH, as expected if the ligand shifts towards the hinge at low pH. The 5,6-DBBt binding is tighter at high than at low pH, suggesting a higher contribution of the interaction with K68 to the ligand binding.

However, except for the H/D exchange data, there is limited support for a pH-dependent ligand shift. The most direct evidence for a pH-independent binding pose of 5,6-DBBt away from the hinge comes from the crystallographic data that shows essentially superimposable ligand binding modes backed up by anomalous scattering data. A crystal contact-induced artifact is implausible because the ligand-binding pocket is not close to a crystallographic interface. Instead, the largely pH-independent ligand pose is likely due to perturbations of the pKa of 5,6-DBBt in the ligand-binding pocket.

Global analysis of ITC data collected for 5,6-DBBt in various buffers in the pH range of 6.5–8.7 (Fig. 4) unequivocally shows that the apparent pK_a_ for dissociation of the triazole proton is lowered by almost one unit upon binding to hCK2α (6.1 vs. 6.93, respectively). Consequently, the inflection point observed for 5,6-DBBt in nanoDSF and ITC experiments must be assigned directly to the dissociation of the ligand triazole proton enforced by ligand binding. No such pH dependence is observed in nanoDSF data obtained for non-dissociable neutral ligands substituted with a methyl group at the triazole nitrogen, all of which bind substantially weaker to the target protein. These results might explain the observed apparent discrepancy between HDX-MS and crystallography. It is possible that at pH 6.7 in the HDX experiment, a small fraction of the neutral form of the ligand might be observed; however, it is not substantial enough to be visible in crystallography, even at lower pH. Based on the pK_a_, the ligand should be predominantly neutral at the lowest tested pH (5.5), yet we still see the pH-independent pose close to K68. The latter could either indicate that the pH in the crystals varies slightly from the pH in the crystallization buffer or that even a hydrogen bond suffices to outcompete halogen bonding.

To put the data on 5,6-DBBt into perspective, we have studied all available structures of benzotriazole resembling compounds in complex with protein kinases (Suppl. Table S4). According to this analysis, ionizable benzotriazole analogues tend to bind close to the lysine, if there are only two halogen substituents, and closer to the hinge, when there are three or four substituents. The non-ionizable analogues, which cannot form a salt bridge, adopt almost universally poses close to the hinge. Hence, it appears that the hydrophobic effect and halogen bonding dominate when there are three or more halogens, or when only a hydrogen bond, but not a salt bridge, would be possible on the side of the lysine. By contrast, the salt bridge dominates in the case of 5,6-DBBt with only two halogen substituents.

Summarizing, thermodynamic studies demonstrate that at a pH range of 6.0 to 8.5, 5,6-DBBt is mostly or at least partly anionic in complex with hCK2α due to binding-induced pKa perturbations from 6.9 in solution down to 6.1 in the complex. Therefore, halogen bonding is outcompeted by salt bridge formation, even at pH below pKa. Hydrogen deuterium exchange data indicate that the hinge region is better protected at pH 6.7 than 8.7, suggesting that a minor population of 5,6-DBBt, not detectable by X-ray crystallography, may shift towards the hinge region, promoting halogen bonding. Despite this subtle effect, ionic interactions predominate over halogen bonding in the hCK2α-5,6-DBBt complex.

## Materials and methods

This manuscript does not involve the use of any animal or human sample/data.

### Expression and purification of hCK2α

The catalytic subunit of human CK2 (hCK2α) was expressed and purified as described previously^35^. Protein sample homogeneity was routinely confirmed by gel electrophoresis. Proper folding of hCK2α was validated with a thermal profile of fluorescence-monitored protein stability prior to calorimetric experiments^34^.

### Ligand synthesis

5,6-dibromo-1*H*-benzotriazole (5,6-DBBt) and 4,5,6,7-tetrabromo-1H-benzotriazole (TBBt) were synthesized according to the previously used methods^35^.

Methylation of 5,6-dibromo-1*H*-benzotriazole: The solution of 5,6-dibromo-1*H*-benzotriazole (300 mg, 1.08 mmol, 1.0 equiv.) in acetonitrile (5 ml), was mixed with DBU (0.26 ml, 1.73 mmol, 1.6 equiv.) and methyl iodide (0.32 ml, 5.20 mmol, 4.8 equiv.) and stirred at 55 °C for 24 h. The volatiles were then evaporated under reduced pressure, and the resulting oily residue was purified by column chromatography using a toluene/dichloromethane 1:1 v/v mixture, followed by crystallization from nitromethane. The yield of 5,6-dibromo-1-methyl-1*H*-benzotriazole (1-CH_3_-5,6-DBBt) was 204 mg (65%, more polar), white solid, m.p. = 208.8–210.8 °C. The yield of 5,6-dibromo-2-methyl-1*H*-benzotriazole (2-CH_3_-5,6-DBBt) was 85 mg (27%, less polar), white solid, m.p. = 200.1–201.9 °C.

**Figure 7:**
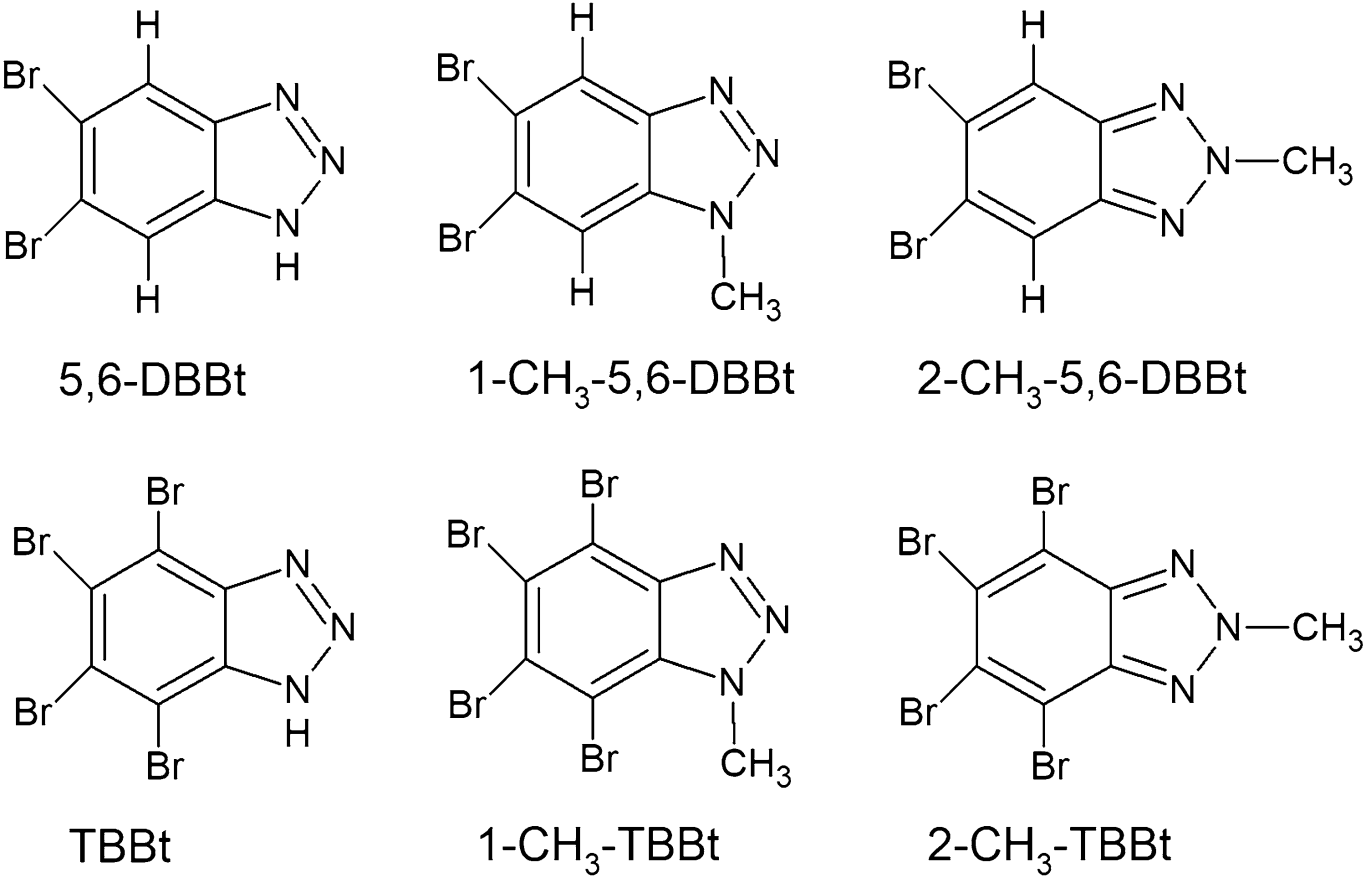
Schematic diagrams of the compounds used in the study.

Methylation of 4,5,6,7-tetrabromo-1*H*-benzotriazole: The solution of 4,5,6,7-tetrabromo-benzotriazole (870 mg, 2.00 mmol, 1.0 equiv.) in acetonitrile (16 ml) was mixed with DBU (0.48 ml, 3.20 mmol, 1.6 equiv.) and methyl iodide (0.64 ml, 9.60 mmol, 4.8 equiv.) and stirred at 55 °C for 24 h. The precipitate was filtered off under reduced pressure and purified by column chromatography using toluene/chloroform 1:1 v/v mixture, followed by crystallization from nitromethane. The yield of 4,5,6,7-tetrabromo-1-methyl-1*H*-benzotriazole (1-CH_3_-TBBt) was 314 mg (35%, more polar), white solid, m.p. = 225.0–226.0 °C. The yield of 4,5,6,7-tetrabromo-2-methyl-1*H*-benzotriazole (2-CH_3_-TBBt) was 50 mg (11%, less polar), white solid, m.p. = 250.0–253.0 °C. The schematic diagrams of the compounds are presented in Fig. 7.

### Crystallization

Crystallization of hCK2α-bromobenzotriazole complexes was carried out as described previously^65^. The protein concentrated to 4–8 mg/ml in the buffer containing 25 mM Tris–HCl pH 8.5, 0.5 M NaCl, and 5 mM β-mercaptoethanol was mixed with 0.25 M ligand DMSO solution in a 1:24 molar ratio. Crystals were grown from 1:1 mixtures of the protein–ligand solution and crystallization buffers listed in Suppl. Material. No additional cryo-protection was used prior to flash-freezing in liquid nitrogen. Diffraction data were collected at the P11 and P14 beamlines of (EMBL) DESY (Hamburg, Germany) and MX beamline 14.1 of BESSY (Berlin, Germany).

### Structure determination

The structure determination was performed as in^65^ for consistency between different pH values. Briefly, the structures were solved by molecular replacement using the structure of hCK2α (PDB code 3WAR) as the search model^75^. They were rebuilt with ARP/wARP^76^ and refined with REFMAC^77^. The R_free_ reflections were selected in thin resolution shells. The structures determined at pH 7.5 were published before^65^. The presence and position of the ligands were verified using anomalous difference maps generated with the Fourier transform of the hCK2α structures with the ligands omitted (Suppl. Figs. S3–S6). The ligand constraints were generated with the PRODRG server^78^. Table 3 summarizes data collection and refinement statistics. The atomic coordinates of the final models and the corresponding structure factors have been deposited in the Protein Data Bank (PDB) with the following accession codes: 5,6-DBBt at pH 5.5—7QGC, at pH 6.5—7QGB, at pH 8.5—7QGD, TBBt at pH 8.5—7QGE.

### Low-volume differential scanning fluorimetry (nanoDSF)

The assay was carried out in 25 mM Bis–Tris Propane, 0.5 M NaCl buffer with constant protein and ligand concentrations of 2.5 μM and 25 μM, respectively. The samples were loaded into nanoDSF Grade Standard Capillaries (NanoTemper Technologies) and analyzed using the Prometheus NT.48 nanoDSF device (NanoTemper Technologies). All experiments were performed and analyzed as described previously^65^.

### Isothermal titration calorimetry (ITC)

ITC measurements were carried out using MicroCal iTC200 (Malvern). In all experiments, hCK2α samples were transferred to the appropriate buffer using Pierce™ Polyacrylamide Spin Desalting Columns (Thermo Scientific). Stock ligand DMSO solutions were diluted with the appropriate DMSO volume before mixing with the buffer to obtain the required ligand concentration with a final DMSO content of 1%. The experimental setup and the analysis algorithm have already been described in detail^65^, the buffer list is presented in Suppl. Material.

### Numeric analysis of nanoDSF and ITC data

Appropriate models were fitted to the experimental data using the Levenberg–Marquardt algorithm implemented in the Origin package (ver. 9.9, www.originlab.com). The standard errors for the derived parameters were estimated according to the Error Propagation formula (https://www.originlab.com/doc/origin-help/nlfit-theory).

### Hydrogen–deuterium exchange mass spectrometry (HDX-MS)

The general experimental procedure was described previously^79^ with the additional sample digestion with 5 μl of protease Type XIII from *Aspergillus saitoi* (30 μg/μl stock) for 30 s on ice before loading onto an immobilized pepsin column. We used 40 μM stock solutions of hCK2α, hCK2α peptides were obtained at pH 8.0, and hydrogen–deuterium exchange experiments were performed for free hCK2α protein and hCK2α-5,6-DBBt complex (tenfold excess of the ligand) at two pH values (6.7 and 8.7).

For 37 hCK2α–derived peptides with extreme HDX protection (indicated in gray in Suppl. Fig. S7), the theoretical percentage of deuteration, D, was calculated by omitting the back-exchange, and the simplified formula $D[\%]= \frac{{M}_{ex}}{\left({n}_{aa}-1-{n}_{pro}\right)}\cdot100\%$ was used instead of the standard one: $D[\%]=\frac{({M}_{ex}-{M}_{ex}^{0})}{({M}_{ex}^{100}-{M}_{ex}^{0})}\cdot100\%$

where n_aa_ and n_pro_ are numbers of all amino acids and proline residues in a peptide. ${M}_{ex}$, ${M}_{ex}^{0}$, and ${M}_{ex}^{100}$ are 10-s, minimal and maximal exchange for a given peptide. Errors were estimated as standard deviations of three independent experiments.

## Supplementary Information

Supplementary Information.

## Data Availability

The coordinates and structure factors have been deposited to the Protein Data Bank (https://www.rcsb.org) under accession numbers: 7QGC (https://www.rcsb.org/structure/7QGC), 7QGB (https://www.rcsb.org/structure/7QGB), 7QGD (https://www.rcsb.org/structure/7QGD), 7QGE (https://www.rcsb.org/structure/7QGE). All other datasets generated and/or analyzed within this study are available from the corresponding author on a reasonable request.

## Abbreviations

hCK2α: Alpha subunit of human casein kinase CK2
TBBt: Tetrabromobenzotriazole
5,6-DBBt: 5,6-Dibromobenzotriazole
TBBi: Tetrabromobenzimidazole
nanoDSF: Low volume differential scanning fluorimetry
ITC: Isothermal titration calorimetry
HDX: Hydrogen–deuterium exchange
MS: Mass spectrometry
MX: Macromolecular X-ray crystallography
DRB: 5,6-Dichloro-1-β-d-ribofuranosylbenzimidazole
DBU: 1,8-Diazabicyclo[5,4,0]undec-7-ene
