# Supplementary material for: Competition between electrostatic interactions and halogen bonding in the protein–ligand system: structural and thermodynamic studies of 5,6-dibromobenzotriazole-hCK2α complexes
Source: Sci Rep. 2022 Nov 8;12:18964. doi: 10.1038/s41598-022-23611-0 (PMC9641685; doi:10.1038/s41598-022-23611-0)
Supplement: Supplementary file 1 — Supplementary Information. [file 41598_2022_23611_MOESM1_ESM.pdf]

Supplementary material for:

Competition between electrostatic interactions and halogen bonding in the protein-ligand system: structural and thermodynamic studies of 5,6-dibromobenzotriazole - hCK2α complexes.

Maria Winiewska-Szajewska[1,2*], Honorata Czapinska[1,3], Magdalena Kaus-Drobek[1], Anna Fricke[1,3], Kinga Mieczkowska[1], Michał Dadlez[1], Matthias Bochtler[1,3], Jarosław Poznański[1*].

[1]Institute of Biochemistry and Biophysics PAS, Pawinskiego 5a, 02-106 Warsaw, Poland
[2]Division of Biophysics, Institute of Experimental Physics, University of Warsaw, Pasteura 5, 02-089 Warsaw, Poland;
[3]International Institute of Molecular and Cell Biology, Trojdena 4, 02-109 Warsaw, Poland

*Correspondence to be addressed to:
e-mail: jarek@ibb.waw.pl
e-mail: mwin@ibb.waw.pl

Supplementary Materials

Buffers:

Buffers used for crystallization:

Protein buffer:
25 mM Tris-HCl pH 8.5, 0.5 M NaCl, 5 mM β-mercaptoethanol

Reservoir buffers:
20 mM sodium formate, 20 mM ammonium acetate, 20 mM sodium citrate tribasic dihydrate, 20 mM sodium potassium tartrate tetrahydrate, 20 mM sodium oxamate, 20% polyethylene glycol 550 monomethyl ester, 10% polyethylene glycol 20 000, and 0.1 M buffering solution of either:

imidazole/MES pH 5.5
imidazole/MES pH 6.5
sodium HEPES/MOPS pH 7.5
Tris/Bicine pH 8.5
CAPSO/NaOH pH 9.5

Buffers used in ITC experiments:

25 mM Bis-Tris Propane 0.5 M NaCl and 1% DMSO; pH 6.5-8.7
25 mM MOPS, 0.5 M NaCl and 1% DMSO; pH 6.5 and 6.7
25 mM MES, 0.5 M NaCl and 1% DMSO; pH 6.5 and 6.7
25 mM BES, 0.5 M NaCl and 1% DMSO; pH 6.5, 6.7 and 7.0
25 mM PIPES, 0.5 M NaCl and 1% DMSO; pH 6.5, 6.7, 7.0 and 7.5
25 mM Tris-HCl, 0.5 M NaCl and 1% DMSO; pH 7.0, 7.5, 8.0, 8.5 and 8.7
25 mM HEPES, 0.5 M NaCl and 1% DMSO; pH 7.0, 7.5 and 8.0
25 mM Tricine, 0.5 M NaCl and 1% DMSO; pH 8.0, 8.5 and 8.7
25 mM TES, 0.5 M NaCl and 1% DMSO; pH 8.0
25 mM CHES, 0.5 M NaCl and 1% DMSO; pH 8.7
25 mM AMPSO, 0.5 M NaCl and 1% DMSO; pH 8.5 and 8.7

Buffers used in HDX experiments:

H_2O Reaction buffer: 25 mM Tris-HCl, 0.5 M NaCl
H_2O Stop Buffer: 2 M glycine buffer, pH 2.5
D_2O Reaction buffer: 25 mM Tris-DCl, 0.5 M NaCl pH 6.7 and pH 8.7
D_2O Stop Buffer: 2 M glycine buffer, pH 2.5

Supplementary Tables

Table S1. Thermodynamic parameters of the 5,6-DBBt affinity to hCK2α determined with the aid of isothermal titration calorimetry (ITC) at different conditions. The data were collected in two or more independent experiments that were separately evaluated.

pH	Buffer	$\Delta G [kJ \cdot mol^{-1}]$	K_d[nM]	$\Delta H [kJ \cdot mol^{-1}]$	$\Delta S [J \cdot mol^{-1} \cdot K^{-1}]$
6.5	Bis-Tris Propane	-39.1±0.4	143±24	-57.6±1.4	-62±6
		-38.8±0.9	158±57	-57.4±4.1	-62±16
		-38.3±0.4	197±30	-55.6±1.4	-58±6
6.7		-40.1±0.5	93±20	-65.0±1.9	-83±8
		-39.3±0.7	128±36	-59.2±2.4	-67±10
		-39.7±0.7	111±31	-51.1±1.9	-38±8
7.0		-40.6±0.3	76±9	-56.2±0.8	-52±4
		-40.8±0.3	72±9	-59.1±0.9	-61±4
7.2		-40.5±0.7	80±24	-60.4±2.4	-67±10
		-40.2±0.6	89±22	-58.2±1.9	-60±8
7.5		-40.4±0.5	83±15	-54.2±1.3	-46±6
		-41.4±0.3	57±6	-57.0±0.7	-52±3
7.7		-41.8±1.3	48±25	-51.7±4.0	-33±17
		-41.5±0.6	54±14	-53.8±1.4	-41±7
8.0		-41.6±0.4	51±8	-53.8±1.1	-41±5
		-41.6±0.6	51±11	-53.8±1.9	-41±8
8.2		-41.9±0.3	46±5	-55.7±0.6	-46±3
		-42.1±0.2	43±3	-54.4±0.4	-41±2
8.5		-42.5±0.5	36±7	-53.1±1.2	-36±5
		-41.9±0.5	46±8	-52.2±1.1	-35±5
8.7		-41.6±0.3	51±5	-55.2±0.5	-46±3
		-41.5±0.3	54±6	-53.2±0.6	-39±3
6.5	MES	-39.7±0.5	110±24	-44.3±1.6	-15±7
		-39.7±0.7	112±30	-42.8±2.0	-11±9
6.5	MOPS	-39.3±0.4	130±22	-46.8±1.3	-25±6
		-38.5±0.4	182±32	-47.0±1.4	-28±6
6.5	BES	-40.7±0.8	74±25	-51.0±2.7	-35± 12
		-39.8±0.6	107±25	-49.2±1.8	-31±8
6.5	PIPES	-38.9±0.9	151±56	-46.3±3.5	-7±4
		-39.4±0.8	126±39	-39.5±2.0	0±9
6.7	MES	-38.6±0.3	173±24	-48.8±1.2	-34±5
		-39.5±1.7	118±8	-44.6±6.6	-17±27
6.7	MOPS	-40.3±0.5	88±19	-49.5±1.3	-31±6
		-40.0±0.9	98±36	-52.3±3.0	-34±12
6.7	BES	-39.5±0.3	121±14	-53.1±0.9	-46±4
		-40.3±0.7	87±24	-52.2±2.1	-40±9
6.7	PIPES	-40.2±1.1	89±38	-34.5±2.6	-11±12
		-39.0±0.9	148±53	-46.1±2.8	-24±12
7.0	Tris	-41.5±0.5	54±11	-57.2±1.7	-53±7
		-40.9±0.4	67±12	-58.4±1.3	-59±6
7.0	PIPES	-40.0±0.4	98±16	-43.9±1.0	-13±4
		-41.9±0.7	45±12	-43.7±1.2	-6±6

7.0	HEPES	-40.7±0.4<br>-41.5±0.5	75±12<br>54±10	-46.8±0.9<br>-48.0±1.1	-20±4<br>-22±5
7.0	BES	-40.6±0.5<br>-40.3±0.8	77±15<br>87±27	-46.7±1.2<br>-50.0±2.5	-20±5<br>-33±11
7.5	Tris	-41.7±0.3<br>-42.0±0.6	49±6<br>43±10	-58.4±0.8<br>-58.6±1.6	-56±4<br>-55±7
7.5	PIPES	-41.7±0.4<br>-41.6±0.5	50±8<br>52±11	-52.4±0.9<br>-50.4±1.2	-36±4<br>-30±5
7.5	HEPES	-42.2±0.4<br>-40.5±0.6	41±6<br>79±20	-52.0±0.8<br>-53.5±2.1	-36±4<br>-44±9
7.5	BES	-40.9±0.3<br>-41.9±0.4	70±8<br>46±7	-53.4±0.7<br>-55.0±0.9	-42±3<br>-44±4
8.0	Tris	-41.7±0.4<br>-43.2±0.4	50±7<br>27±5	-57.4±1.1<br>-58.5±1.2	-53±5<br>-51±5
8.0	Tricine	-42.0±0.4<br>-42.1±0.4	43±6<br>42±7	-52.1±0.8<br>-55.2±1.0	-34±4<br>-44±5
8.0	HEPES	-41.1±0.6<br>-41.8±0.5	62±15<br>48±10	-54.5±1.9<br>-55.6±1.4	-45±8<br>-46±6
8.0	TES	-41.3±0.3<br>-41.3±0.4	59±6<br>57±10	-55.7±0.6<br>-57.3±1.3	-48±3<br>-53±6
8.5	Tris	-41.8±0.6<br>-42.4±0.6	48±12<br>37±9	-51.8±1.7<br>-52.6±1.4	-34±8<br>-34±6
8.5	Tricine	-42.8±0.7<br>-42.7±0.5	32±9<br>33±7	-51.3±1.4<br>-51.3±1.0	-29±7<br>-29±5
8.5	AMPSO	-43.8±0.6<br>-41.9±0.5	22±5<br>45±10	-51.0±1.0<br>-51.9±1.1	-24±5<br>-33±5
8.7	Tris	-41.3±0.5<br>-41.2±04	59±11<br>59±10	-53.1±1.1<br>-55.2±1.1	-40±5<br>-47±5
8.7	Tricine	-41.2±0.4<br>-41.5±0.5	61±10<br>54±11	-53.9±1.0<br>-54.0±1.4	-43±5<br>-42±6
8.7	AMPSO	-42.1±0.5<br>-42.8±0.8	43±9<br>31±11	-51.6±1.1<br>-52.6±2.1	-32±5<br>-33±9
8.7	CHES	-43.3±1.0<br>-42.0±0.5	26±10<br>43±9	-55.0±2.6<br>-51.9±1.2	-39±11<br>-33±5
1-CH_3-5,6-DBBt		-33.2±1.0<br>-32.9±1.7	1520±600<br>1710±1200	-7.0±0.8<br>-7.8±2.3	88±5<br>84±13

Table S2. Data collection statistics for all crystals of the presented complexes. Published data [2] are shaded in red, data deposited in the PDB for the current paper – in gray, the unpublished repetitions are in white.

	hCK2 - 5,6-DBBt	hCK2 - 5,6-DBBt	hCK2 - 5,6-DBBt	hCK2 - 5,6-DBBt	hCK2 - 5,6-DBBt	hCK2 - 5,6-DBBt	hCK2 - 4,5,6,7-TBBt	hCK2 - 4,5,6,7-TBBt	hCK2 - 4,5,6,7-TBBt	hCK2 - 4,5,6,7-TBBt	hCK2 - 4,5,6,7-TBBt
pH	5.5	6.5	7.5	7.5	7.5	8.5	7.5	7.5	7.5	8.5	8.5
Inhibitor	cocryst.	cocryst.	cocryst.	cocryst.	soaked	soaked	cocryst.	cocryst.	soaked	soaked	soaked
Mg^{2+}	4 mM	4 mM	4 mM	-	4 mM	4 mM	-	-	4 mM	4 mM	4 mM
2nd binding site	no	yes (weak)	no	yes (2 conf.)	yes (weak)	yes	yes	yes	yes (weak)	yes (very weak)	yes (very weak)
Space group	$P4_22_12$	$P4_22_12$	$P4_22_12$	$P4_32_12$	$P4_22_12$	$P4_22_12$	$P4_22_12$	$P4_22_12$	$P4_22_12$	$P4_22_12$	$P4_22_12$
Cell dimensions											
a,b (Å)	127.5	128.5	127.2	128.4	128.1	129.5	127.4	128.5	128.1	127.8	127.7
c (Å)	61.0	61.2	60.9	124.2	61.0	60.9	61.0	61.3	61.3	61.2	61.3
Wavelength (Å)	0.9117	0.9117	0.9117	0.9150	0.9117	0.9116	0.9116	0.9116	0.9117	0.9117	0.9117
Beamline	DESY P11	DESY P11	DESY P11	BESSY 14.1	DESY P11	DESY P11	DESY P14	DESY P14	DESY P11	DESY P11	DESY P11
Resolution (Å) lowest shell highest shell	2.55 (45.1 – 7.55) (2.70 - 2.55)	2.58 (45.4 – 7.64) (2.73 - 2.58)	1.93 (36.2 – 5.72) (2.04 – 1.93)	2.14 (45.4 - 6.36) (2.27 - 2.14)	2.25 (45.3 - 6.68) (2.38 - 2.25)	2.30 (45.8 – 6.84) (2.44 – 2.30)	1.88 (45.0-5.60) (1.99 - 1.88)	1.95 (20 – 5.83) (2.07 - 1.95)	2.68 (45.3 – 7.92) (2.84 - 2.68)	2.27 (45.2 – 6.74) (2.41 – 2.27)	2.25 (45.1 – 6.68) (2.39 - 2.25)
R_{meas} (%)*	30.4 (9.4, 135.9)	31.4 (6.2, 210.5)	14.2 (3.8, 170.3)	13.6 (3.8, 176.4)	24.7 (5.3, 187.2)	11.2 (3.9, 87.3)	9.1 (5.5, 127.8)	11.0 (5.7, 150.2)	24.1 (5.2, 187.6)	11.5 (3.6, 174.9)	15.5 (4.0, 200.3)
$CC_{1/2}$*	99.5 (99.9, 80.3)	99.6 (99.9, 68.6)	99.9 (100, 80.7)	100 (100, 71.0)	99.8 (100, 72.4)	99.9 (100, 93.5)	99.9 (99.9, 85.1)	99.9 (99.9, 83.9)	99.8 (99.9, 72.3)	100 (100, 84.5)	99.9 (100, 76.9)
I/σI*	10.7 (31.7, 2.03)	12.6 (43.4, 1.99)	22.7 (82.5, 1.98)	22.4 (69.1, 2.03)	15.4 (56.5, 1.98)	27.7 (80.2, 4.24)[$]	20.6 (51.1, 2.07)	18.5 (49.2, 2.06)	15.2 (56.1, 2.03)	26.1 (88.4, 2.53)[$]	21.7 (77.8, 2.00)
Completeness (%)*	99.9 (99.3, 99.8)	99.8 (99.3, 99.8)	99.9 (99.4, 99.5)	99.9 (99.3, 99.7)	99.8 (99.5, 99.1)	99.2 (99.5, 99.4)	99.6 (99.7, 97.6)	98.9 (98.8, 94.4)	99.4 (99.1, 99.5)	99.8 (99.5, 99.5)	99.7 (99.5, 98.5)
Multiplicity*	26.3 (23.0, 26.9)	26.0 (25.3, 26.4)	26.2 (23.3, 25.4)	26.7 (23.0, 27.3)	26.3 (23.1, 25.3)	25.4 (22.3, 26.1)	24.8 (21.3, 23.6)	24.6 (21.7, 23.5)	25.9 (22.0, 25.2)	26.3 (21.9, 26.5)	26.2 (22.6, 26.2)
Number of reflections	16932	16669	38226	57785	24732	23379	41198	37408	14815	24013	24556

* Lowest and highest shell in brackets. [$] Data cut due to an ice ring.

Table S3. Characterization of short halogen…oxygen contacts identified in the newly determined structures.

PDB	pH	XB donor	XB acceptor	Br…Acc distance (Å)	C-Br…Acc angle (deg)
7QGC	8.5	Br5 (DBBt)	O (E114)	3.95	161
7QGC	8.5	Br6 (DBBt)	OD1 (N 118)	4.44	139
7QGB	6.5	Br5 (DBBt)	O (E114)	4.03	161
7QGB	6.5	Br6 (DBBt)	OD1 (N 118)	4.57	142
7QGD	5.5	Br5 (DBBt)	O (E114)	4.05	159
7QGD	5.5	Br6 (DBBt)	OD1 (N 118)	4.47	147
7QGE	8.5	Br4 (TBBt)	O(V116)	3.08	169
7QGE	8.5	Br5 (TBBt)	O(E114)	3.94	163
7QGE	8.5	Br6 (TBBt)	Ring center (F113)	3.84	160

Table S4. Poses of benzotriazole related compounds in the kinase pockets. The compounds with large modification at position 1 and modified at position 2 have been eliminated, due to the steric hindrance preventing the potential contact with the lysine (K). K - the lysine at the bottom of the pocket corresponding to K68 in hCK2α. pKa^{calc} - predicted pKa of deprotonation with MolGpKa service [1].

PDB	pKa^{exp}/ pKa^{calc}	Predicted 5-mem. ring status at pH 7.5 in solution	Proximity	5-membered ring constitution	Compound	Kinase	Cryst. buffer pH
1P5E	4.78/4.1	charged	hinge	NNN	4,5,6,7-tetrabromobenzotriazole	CDK2	7
1J91	4.78/4.1	charged	K	NNN	4,5,6,7-tetrabromobenzotriazole	mCK2α	8.5
6TLL	4.78/4.1	charged	hinge/K	NNN	4,5,6,7-tetrabromobenzotriazole	hCK2α	7.5
6TLR	5.84/6.4	charged	hinge	NNN	4,7-dibromobenzotriazole	hCK2α	7.5
6TLO	5.91/5.1	charged	K/hinge	NNN	4,5,6-tribromobenzotriazole	hCK2α	7.5
6TLS	6.38/6.3	charged	K/hinge	NNN	4,6-dibromobenzotriazole	hCK2α	7.5
6TLU	6.49/6.1	charged	K	NNN	4,5-dibromobenzotriazole	hCK2α	7.5
6TLP	6.93/6.9	charged	K	NNN	5,6-dibromobenzotriazole	hCK2α	7.5
5TS8	6.93/6.9	charged	K/hinge	NNN	5,6-dibromobenzotriazole	mCK2α	7.5
6TLV	7.55/7.5	charged/ neutral	K	NNN	5-bromobenzotriazole	hCK2α	7.5
6TLW	7.08/7.2	charged	K	NNN	4-bromobenzotriazole	hCK2α	7.5
7A1Z	/4.6	charged	K	NNN	6-bromo-5-chloro-triazolo[4,5-b]pyridine	hCK2α′	8.5
7A1B	/4.8	charged	K	NNN	5,6-dibromo-triazolo[4,5-b]pyridine	hCK2α′	8.5
7A22	/3.6	charged	hinge	NNN	5,6,7-tribromo-triazolo[4,5-b]pyridine	hCK2α′	8.5
7A49	/4.6	charged	K	NNN	6-bromo-5-chloro-triazolo[4,5-b]pyridine	hCK2α	5.5
7A4B	/4.8	charged	K	NNN	5,6-dibromo-triazolo[4,5-b]pyridine	hCK2α	5.5
7A4C	/3.6	charged	hinge	NNN	5,6,7-tribromo-triazolo[4,5-b]pyridine	hCK2α	5.5
3KXG	/6.2	charged	hinge	NNC	3,4,5,6,7-pentabromoindazole	mCK2α	8
3KXN	/7.0	charged	hinge	NCN	4,5,6,7-tetraiodobenzimidazole	mCK2α	8
7A2H	/7.3	charged	hinge	NCN	5,6,7-tribromo-imidazo[4,5-b]pyridine	hCK2α′	8.5
2OXY	/10.9	neutral	hinge	NCN	4,5,6,7-tetrabromobenzimidazole	mCK2α	8
3PVG	/3.2[a]	neutral	hinge	NCN	(4,5,6,7-tetrabromobenzimidazol-1-yl)acetic acid	mCK2α	8
3H30	/13	neutral	hinge	NCN +N1-modified	5,6-dichloro-1-beta-D-ribofuranosylbenzimidazole	hCK2α	5.6
3OFM	/13.6	neutral	hinge	NNN +N1-modified	3-(4,5,6,7-tetrabromobenzotriazol-1-yl)propan-1-ol	hCK2α′	8.5
3RPS	/13.6	neutral	hinge	NNN +N1-modified	3-(4,5,6,7-tetrabromobenzotriazol-1-yl)propan-1-ol	hCK2α	5.0
6HMQ	/13.6	neutral	hinge	NNN +N1-modified	3-(4,5,6,7-tetrabromobenzotriazol-1-yl)propan-1-ol	hCK2α′	8.5
7AT9	/13.6	neutral	hinge	NNN +N1-modified	3-(4,5,6,7-tetrabromobenzotriazol-1-yl)propan-1-ol	hCK2α′	8.5
5OWH		neutral	hinge/K	NCN +N1-modified	3-(4,5,6,7-tetrabromobenzimidazol-1-yl)propan-1-amine	hCK2α	5.5
5OWL		neutral	hinge	NCN +N1-modified	3-(4,5,6,7-tetrabromobenzimidazol-1-yl)propan-1-amine	hCK2α	5.5
3MY1	/13.0;12.9	neutral	hinge	NCN +N1-modified	8-(4,5,6,7-tetrabromobenzimidazol-1-yl]octanoic acid	CDK9	6.5
3MY5	/13.0;12.9	neutral	hinge	NCN +N1-modified	8-(4,5,6,7-tetrabromobenzimidazol-1-yl]octanoic acid	CDK2	7
7ATV	/13.6;13.7	neutral	hinge	NCN +N1-modified	N'-[2-(3,4-dichlorophenyl)ethyl]-N-[4-[4,5,6,7-tetrabromobenzimidazol-1-yl]butyl]butanediamide	hCK2α′	8.5
7AT5	/13.6;13.7	neutral	hinge	NCN +N1-modified	N'-[2-(3,4-dichlorophenyl)ethyl]-N-[4-[4,5,6,7-tetrabromobenzimidazol-1-yl]butyl]butanediamide	hCK2α	6.5
6SPX	/4.7[b]	neutral	hinge	NCN +N1-modified	8-(4,5,6,7-tetrabromobenzimidazol-1-yl)octanoic acid	hCK2α	5.6
6SPW	/4.5[b]	neutral	hinge	NCN +N1-modified	8-(4,5,6,7-tetraiodobenzimidazol-1-yl)octanoic acid	hCK2α	5.6
4FBX	/~3[c]	neutral	hinge	NCN +N1-modified	ARC-1154 (bisubstrate conjugate of (4,5,6,7-tetrabromobenzimidazol-1-yl)acetic acid	hCK2α	5.25
4KWP	/13.0;13.3	neutral	hinge	NCN +N1-modified	4,5,6,7-tetrabromo-1-(2-deoxy-beta-D-erythro-pentofuranosyl)-benzimidazole	hCK2α	8.5

[a] The benzimidazole ring is uncharged but the attached group is charged and forms an ionic pair with K68.
[b] The attached group is a long chain charged at the end.
[c] The attached peptide part is highly charged and reach the protein substrate binding site.

Supplementary Figures

Fig. S1

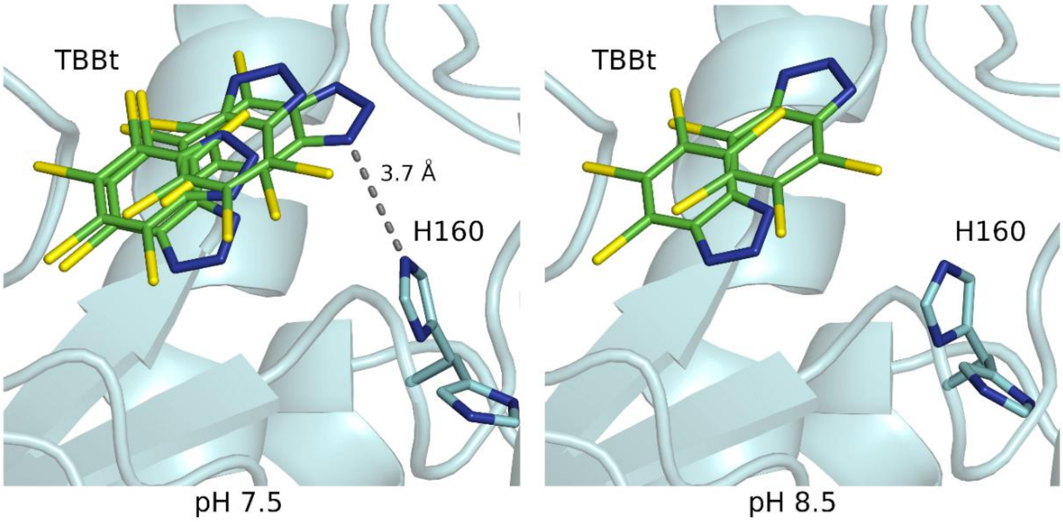

Suppl. Fig. 1. The interaction of TBBt with the ligand binding pocket of hCK2α. The dashed line indicates the proximity of H160 (modeled in two conformations) to the triazole ring of the ligand in one of its four poses at pH 7.5.

Fig. S2

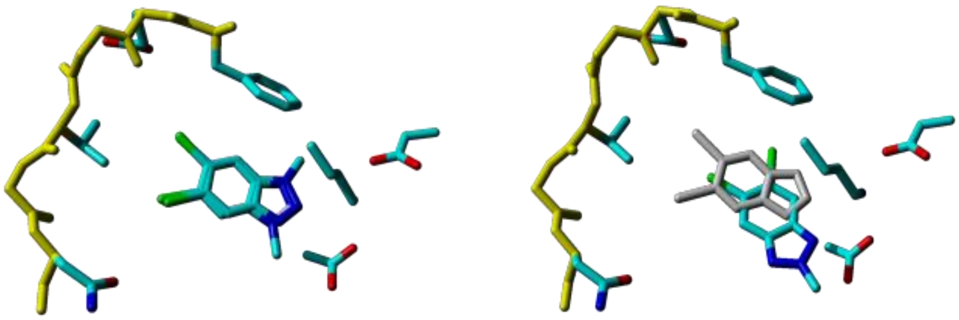

Suppl. Fig. 2. Model of 1CH_3-5,6-DBBt (A) and 2CH_3-5,6-DBBt (B) binding in hCK2α ATP-binding site. Modeling was performed with Yasara Structure package, using yasara2 force-field. Structure of 5,6-DBBt-hCK2α complex (7QGD) was used as the reference and original ligand was substituted with methylated analog. The procedure was restricted to the cuboid region that contained all residues from the ATP binding site. During simulations, the sidechain atoms of protein residues proximal to the location of the original ligand (8 Å threshold) were flexible, while coordinates of all other protein atoms were kept fixed.

Fig. S3

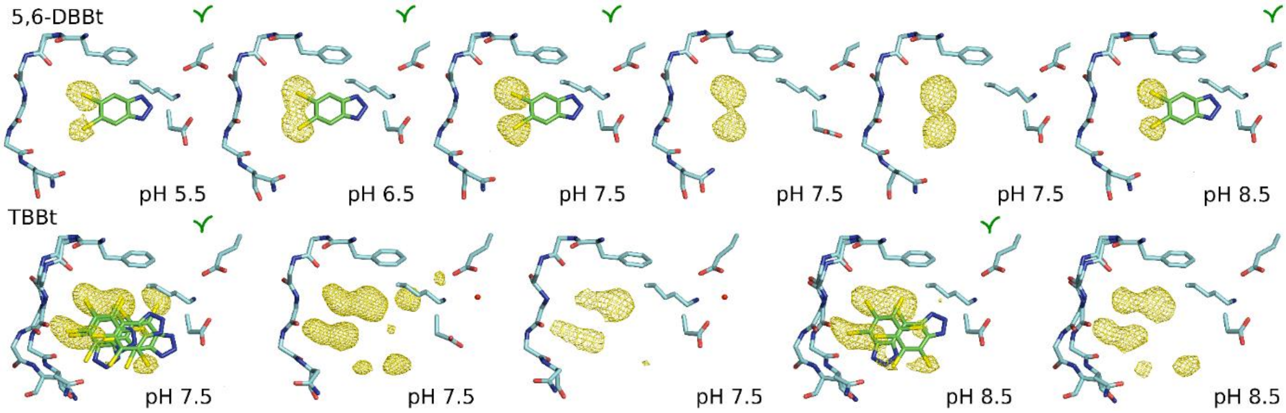

Suppl. Fig. 3. The primary ligand binding site in complexes of hCK2α with 5,6-DBBt (top) and TBBt (bottom) at different pH values. The anomalous difference Fourier maps were calculated for the models without the ligands, contoured at 4.5 rmsd and shown in yellow. The structures obtained at pH 7.5 have been published before (PDB 6TLP and 6TLL) [2]. The replicas indicate that the occupation of the primary binding site is consistent in the crystals. Only the first presented structures at each pH value (mark with ticks) were deposited in the PDB, the replicas were not refined further. The location of the second hinge conformation in complexes with TBBt was ambiguous and traced only tentatively for chain completeness.

Fig. S4

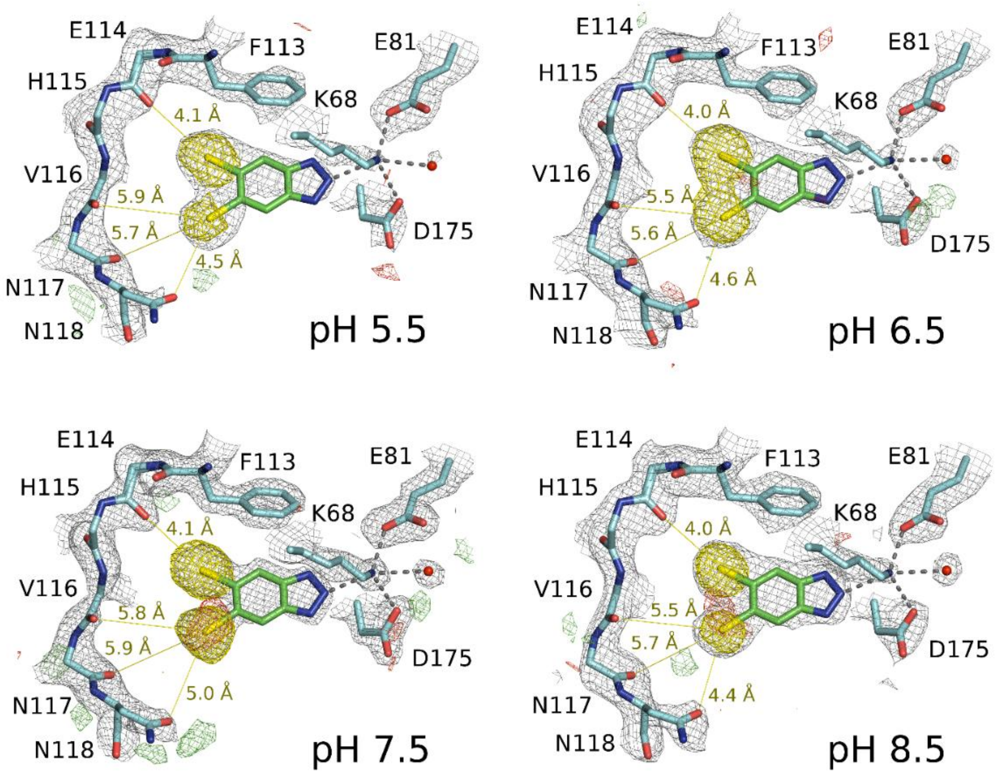

Suppl. Fig. 4. The binding mode of 5,6-DBBt in complex with hCK2α in different pH conditions. The anomalous difference Fourier maps were calculated for the models without the ligands, contoured at 4.5 rmsd and shown in yellow. The composite omit electron density maps are contoured at 1.5 rmsd and shown in gray, the difference density map is contoured at 3 rmsd (green) and -3 rmsd (red). The structure obtained at pH 7.5 has been published before (PDB 6TLP) [2].

Fig. S5

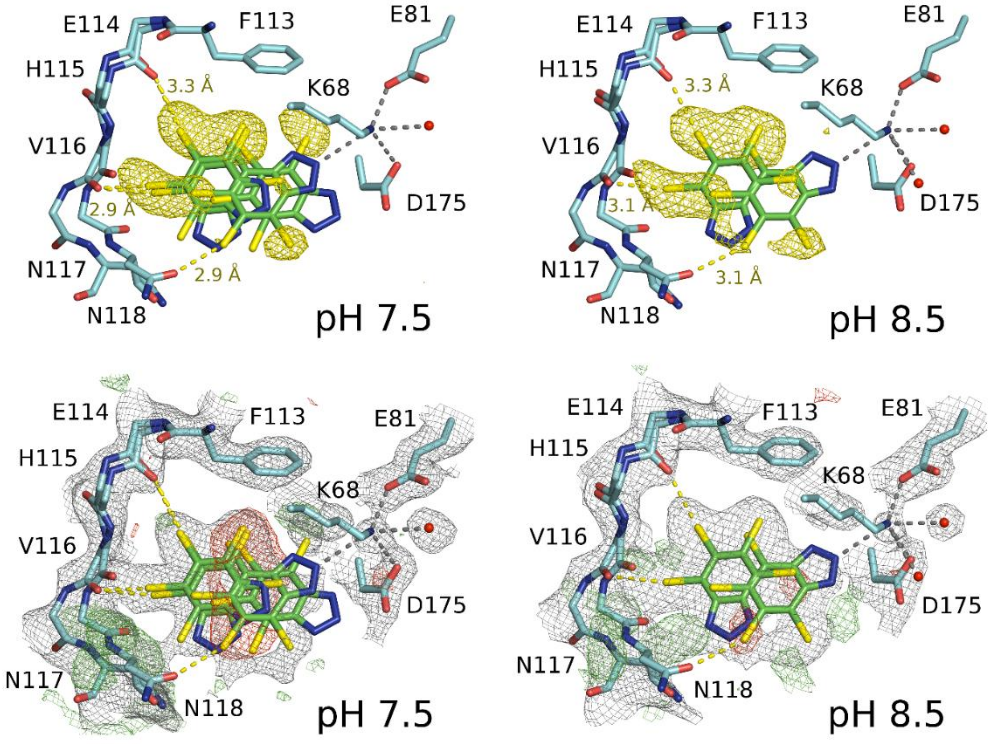

Suppl. Fig. 5. The binding mode of 4,5,6,7-TBBt in complex with hCK2α at two different pH values. The anomalous difference Fourier maps were calculated for the models without the ligands, contoured at 4.5 rmsd and shown in yellow (top panel). The composite omit electron density maps are contoured at 1 rmsd and shown in gray, the difference density map is contoured at 3 rmsd (green) and -3 rmsd (red) (bottom panel). The structure obtained at pH 7.5 has been published before (PDB 6TLL) [2].

Fig. S6

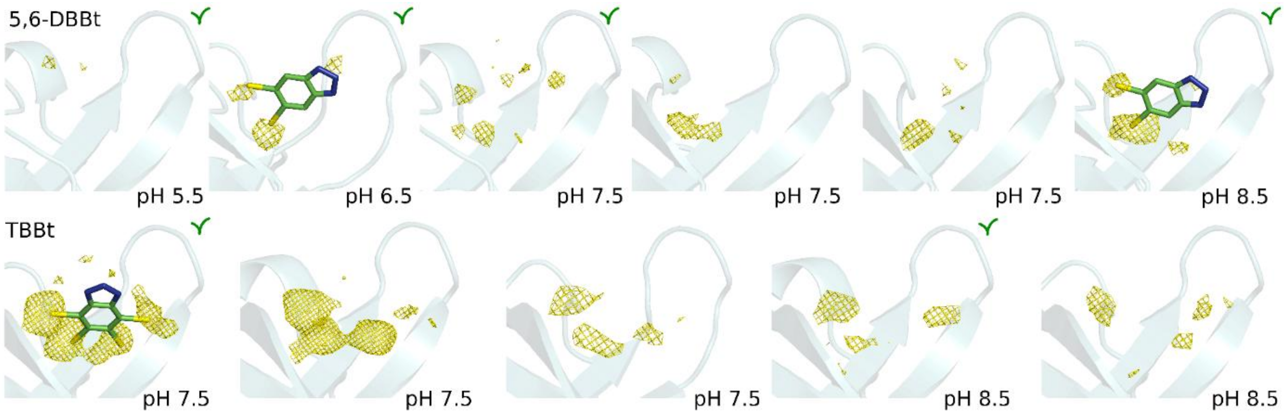

Suppl. Fig. 6. The secondary ligand binding site in complexes of hCK2α with 5,6-DBBt (top) and TBBt (bottom) at different pH values. The anomalous difference Fourier maps were calculated for the models without the ligands, contoured at 2.5 rmsd and shown in yellow. The structures obtained at pH 7.5 have been published before (PDB 6TLP and 6TLL) [2]. The replicas indicate that the occupation of the secondary binding site is inconsistent in the crystals. Only the first presented structures at each pH value (marked with ticks) were deposited in the PDB, the replicas were not refined further. The position of the second ligand molecule in the crystals clashed with the protein in the region of residue 36 and thus the ligands were in most cases modelled at half occupancy only (to match one of the alternative conformations of the protein).

Fig. S7

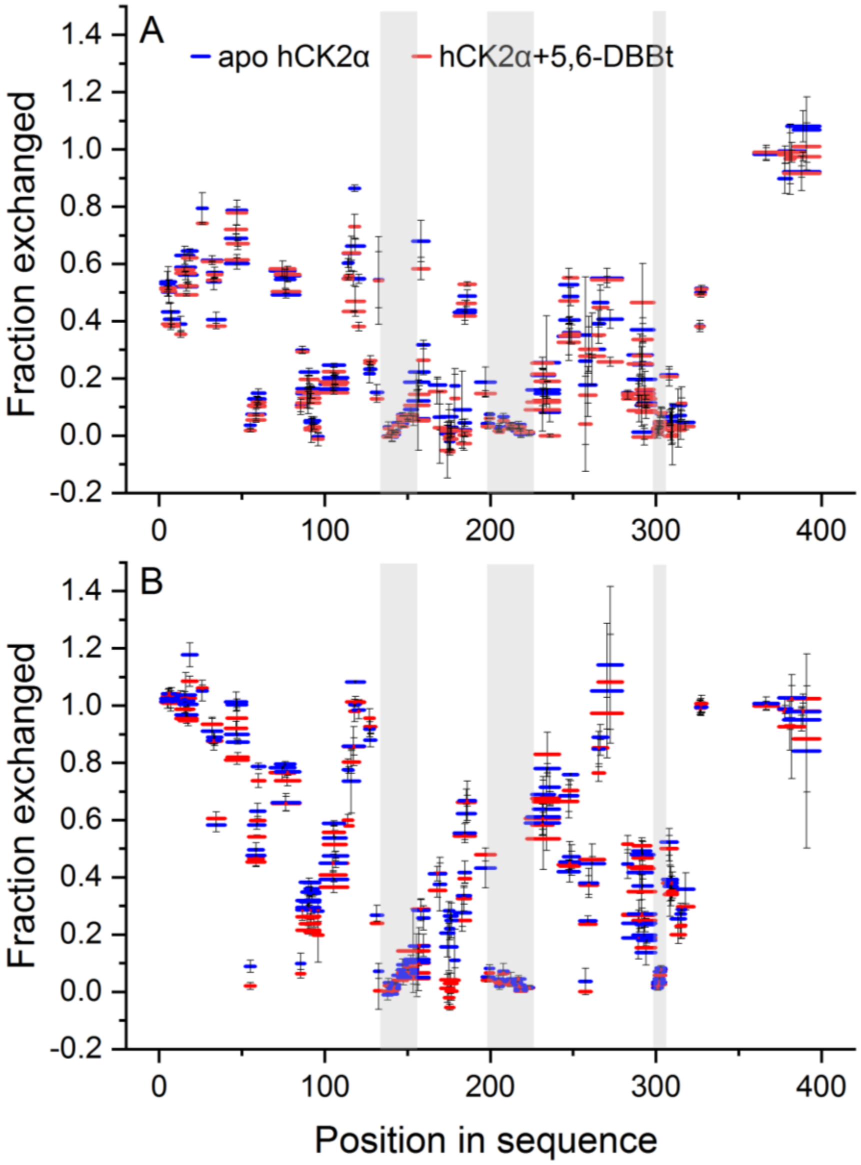

Suppl. Fig. 7. Results of 10 sec hydrogen deuterium exchange determined for hCK2α at pH 6.7 (upper panel) and 8.7 (lower panel) in the free form (red) and in complex with 5,6-DBBt (blue). Peptides for which the theoretical deuteration was calculated are denoted in gray.

References

1. Pan X, Wang H, Li C, Zhang JZH, Ji C. MolGpka: A Web Server for Small Molecule pKa Prediction Using a Graph-Convolutional Neural Network. *Journal of Chemical Information and Modeling*. 2021;61(7):3159-3165.
2. Czapinska H, Winiewska-Szajewska M, Szymaniec-Rutkowska A, Piasecka A, Bochtler M, Poznanski J. Halogen Atoms in the Protein-Ligand System. Structural and Thermodynamic Studies of the Binding of Bromobenzotriazoles by the Catalytic Subunit of Human Protein Kinase CK2. *J Phys Chem B*. Mar 2021;125(10):2491-2503. doi:10.1021/acs.jpcb.0c10264
